# Transcriptome profiling of antiviral immune and dietary fatty acid dependent responses of Atlantic salmon macrophage-like cells

**DOI:** 10.1186/s12864-017-4099-2

**Published:** 2017-09-08

**Authors:** Khalil Eslamloo, Xi Xue, Jennifer R. Hall, Nicole C. Smith, Albert Caballero-Solares, Christopher C. Parrish, Richard G. Taylor, Matthew L. Rise

**Affiliations:** 1 Department of Ocean Sciences, Memorial University of Newfoundland, 1 Marine Lab Road, St. John’s, NL A1C 5S7 Canada; 2 Aquatic Research Cluster, CREAIT Network, Memorial University of Newfoundland, 1 Marine Lab Road, St. John’s, NL A1C 5S7 Canada; 3 Cargill Innovation Center, 4335 Dirdal, Norway

**Keywords:** Microarray, Nutrigenomics, Teleost fish, Omega-3/omega-6 fatty acids, Poly(I:C), Pathogen recognition, FABP4

## Abstract

**Background:**

Due to the limited availability and high cost of fish oil in the face of increasing aquaculture production, there is a need to reduce usage of fish oil in aquafeeds without compromising farm fish health. Therefore, the present study was conducted to determine if different levels of vegetable and fish oils can alter antiviral responses of salmon macrophage-like cells (MLCs). Atlantic salmon (*Salmo salar*) were fed diets containing 7.4% (FO7) or 5.1% (FO5) fish oil. These diets were designed to be relatively low in EPA + DHA (i.e. FO7: 1.41% and FO5: 1%), but near the requirement level, and resulting in comparable growth. Vegetable oil (i.e. rapeseed oil) was used to balance fish oil in experimental diets. After a 16-week feeding trial, MLCs isolated from fish in these dietary groups were stimulated by a viral mimic (dsRNA: pIC) for 6 h (qPCR assay) and 24 h (microarray and qPCR assays).

**Results:**

The fatty acid composition of head kidney leukocytes varied between the two dietary groups (e.g. higher 20:5n-3 in the FO7 group). Following microarray assays using a 44K salmonid platform, Rank Products (RP) analysis showed 14 and 54 differentially expressed probes (DEP) (PFP < 0.05) between the two diets in control and pIC groups (FO5 vs. FO7), respectively. Nonetheless, Significance Analysis of Microarrays (SAM, FDR < 0.05) identified only one DEP between pIC groups of the two diets. Moreover, we identified a large number (i.e. 890 DEP in FO7 and 1128 DEP in FO5 overlapping between SAM and RP) of pIC-responsive transcripts, and several of them were involved in TLR−/RLR-dependent and cytokine-mediated pathways. The microarray results were validated as significantly differentially expressed by qPCR assays for 2 out of 9 diet-responsive transcripts and for all of the 35 selected pIC-responsive transcripts.

**Conclusion:**

*Fatty acid-binding protein adipocyte* (*fabp4*) and *proteasome subunit beta type-8* (*psmb8*) were significantly up- and down-regulated, respectively, in the MLCs of fish fed the diet with a lower level of fish oil, suggesting that they are important diet-responsive, immune-related biomarkers for future studies. Although the different levels of dietary fish and vegetable oils involved in this study affected the expression of some transcripts, the immune-related pathways and functions activated by the antiviral response of salmon MLCs in both groups were comparable overall. Moreover, the qPCR revealed transcripts responding early to pIC (e.g. *lgp2*, *map3k8*, *socs1*, *dusp5* and *cflar*) and time-responsive transcripts (e.g. *scarb1*-*a*, *csf1r*, *traf5a*, *cd80* and *ctsf*) in salmon MLCs. The present study provides a comprehensive picture of the putative molecular pathways (e.g. RLR-, TLR-, MAPK- and IFN-associated pathways) activated by the antiviral response of salmon MLCs.

**Electronic supplementary material:**

The online version of this article (10.1186/s12864-017-4099-2) contains supplementary material, which is available to authorized users.

## Background

Nutritional modulation of fish innate immune responses with different diets (e.g. proteins and amino acids, lipids and fatty acids, carbohydrates, vitamins and minerals) has been well-documented [1, 2]. Fatty acids, notably polyunsaturated fatty acids (PUFAs), play an important role in innate immune responses and the functions of immune cells (e.g. T-cells, B-cells, natural killer cells and macrophages) through various mechanisms (e.g. antigen presentation or phagocytosis) [3, 4]. Dietary omega (n)-3, n-6 or n-9 fatty acids can variably alter (i.e. increase or decrease) the production of ILs (interleukins) and TNF (tumour necrosis factor) as well as the activity (e.g. phagocytosis) and proliferation of leukocytes (e.g. T-cells and macrophages) [3–6]. Indeed, n-3 fatty acids [e.g. eicosapentaenoic acid (EPA, 20:5n-3) and docosahexaenoic acid (DHA, 22:6n-3)] exhibit their inhibitory roles or anti-inflammatory functions through suppressing cytokines (e.g. IL-1b and IL6) and activating anti-inflammatory factors [7]. Importantly, several studies established the EPA- and DHA-dependent suppression of pathogen-associated molecular pattern (PAMP)-induced responses via NFKB (nuclear factor kappa-B) signalling in mammalian macrophages [8, 9]. On the other hand, n-6-derived eicosanoids play pro-inflammatory roles in immune responses [10]. Hence, optimal levels of n-3/n-6 fatty acids contribute to a balanced immune response.

Similar to other vertebrates, fishes must acquire essential polyunsaturated fatty acids (e.g. linoleic acid, linolenic acid, EPA and DHA) from the diet [1]. Aquaculture production has been steadily growing [11], although over-fishing and the dramatic collapse of many marine fish stocks have led to the limited supply of marine ingredients that provide the required long chain n-3 fatty acids in aquafeeds [12, 13]. Hence, there is an increasing trend toward replacing fish oils with vegetable oils in fish diets. Diets containing high levels of vegetable oils may have low levels of some essential n-3 fatty acids (e.g. EPA and DHA) and an unbalanced n-6/n-3 ratio. In mammals, different ratios of dietary n-6/n-3 were shown to change the fatty acid composition of plasma, as well as immune function and macrophage activation [14, 15]. Correspondingly, the consumption of vegetable oil-rich diets can cause some variation in fish immunological responses and resistance to pathogens in a species- and lipid source-dependent manner [1, 16]. For example, there was reduced leukocyte phagocytic activity and increased expression of the *mx* gene (i.e. myxovirus resistance, interferon-inducible gene) in response to viral mimic stimulation in sea bream (*Sparus aurata*) fed soybean and linseed oil containing diets compared to fish oil [17]. Higher levels of vegetable oil in the diet up-regulated the expression of *tlr3* (*toll-like receptor 3*) and *tlr7* in head kidney of polyriboinosinic polyribocytidylic acid (pIC)-injected Atlantic salmon (*Salmo salar*) [18]. On the other hand, Booman et al. [19] reported that camelina oil-containing diets (replacement of 40 or 80% of fish oil with camelina oil) did not change the antiviral immune response of Atlantic cod (*Gadus morhua*) at the transcriptome level.

The production of Atlantic salmon, one of the most economically important aquaculture finfish species, is increasing worldwide [20]. The largest proportion of fish oil used in the global aquaculture industry is consumed by farmed Atlantic salmon [12, 13], but this usage (e.g. forage fish equivalents needed to produce a unit of salmon) has been declining over the last two decades [13]. Still, there is a need to further decrease the level of fish oil in salmon aquafeed, and also to determine if the immune physiology (e.g. antiviral response) of salmon is influenced by lower EPA + DHA intake. Previous studies have demonstrated that the replacement of fish oil with soybean or rapeseed oils does not change the susceptibility of Atlantic salmon to bacterial (*Aeromonas salmonicida*) infection, the phagocytic activity of macrophages, or cytokine (TNF and IL-1B) expression of lipopolysaccharide (LPS)-stimulated head kidney leukocytes (HKLs) in this species [21, 22]. Since Atlantic salmon is susceptible to several viral pathogens [e.g. infectious salmon anaemia virus (ISAV)], it is of paramount importance to determine if replacement of fish oil with vegetable oil in the diet can alter salmon antiviral responses. To address this issue, we used two diets (FO7: 7.4% fish oil; FO5: 5.1% fish oil) which were relatively low in EPA + DHA: 1.4% and 1% of the diet; 4.74% and 3.57% of the fatty acids, respectively. They contained lower EPA + DHA levels compared to a previous study [21], and were close to the EPA + DHA requirement level (4.4% of fatty acids) of Atlantic salmon [23]. Salmon diets formulated by Ruyter et al. [24] with 0 to 2% EPA + DHA as a proportion of diet resulted in a significant non-linear correlation with growth. Their data indicate the growth response maximises around 1.25% EPA + DHA as a proportion of the diet, which is near the middle of our formulated range. When measured, the proportion of EPA + DHA in our diet FO5 was 3.57% of total fatty acids, and in diet FO7 it was 4.74%, which represents an increase of a third. Growth data in two long-term trials by Rosenlund et al. [23] suggest Atlantic salmon require dietary EPA + DHA at 2.7 to 4.4% of total fatty acids. Our diet fatty acid proportions are situated on both sides the 4.4% value. The low EPA + DHA diets used in the current experiment were associated with comparable growth performance of salmon and may be regarded as practical diets for salmon farming. We used rapeseed oil as the vegetable oil source in the present study. Rapeseed oil is one of the most suitable candidates for fish oil substitution in Atlantic salmon feed since it contains n-3 fatty acids (~7%) and high levels of monounsaturated fatty acids (~63% MUFA) that increase its resistance to oxidation and provide the required energy for fish [25–27].

Macrophages play key roles in innate immune responses of fish through pathogen recognition, cytokine production and phagocytosis [28], and their functions can be greatly affected by dietary fatty acids [6]. Microarray analyses may be used to assess global gene expression changes associated with immunological responses [29], yielding a comprehensive picture of molecular pathways activated by an immune stimulus in cells. Microarray analyses were previously employed to profile the transcriptome responses of salmon macrophage-like cells to ISAV infection [30, 31]. The present study aimed to characterise the transcriptome and physiological response of Atlantic salmon macrophage-like cells to a viral mimic, and the immunomodulatory effect of low dietary EPA + DHA on these cells, using microarrays, real-time quantitative polymerase chain reaction (qPCR), fatty acid analysis, and cellular assays (e.g. phagocytosis).

## Methods

### Fish and experimental diets

Two diets (5 mm pellets) with different levels of fish oil (i.e. FO7: 7.4% and FO5: 5.1% of the diet), and therefore different levels of DHA and EPA (i.e. FO7: 1.41% and FO5: 1.00% of the diet), were formulated and produced by EWOS [EWOS Innovation (now Cargill Innovation Center), Dirdal, Norway] for use in this study (Table 1).

**Table 1:** The composition of experimental diets

Ingredients	omega-3LC1.4 (FO7) %	omega-3LC1 (FO5) %
Fish meal	5	5
Animal byproduct	21.6	21.4
Vegetable protein	33.3	33.8
Fish oil	7.4	5.1
Vegetable oil	19.9	22.1
Binder	10.4	10.4
Premix	2.4	2.4
EPA + DHA content	1.41	1
Fatty acids %^a^		
14:0	1.96 ± 0.011	1.42 ± 0.011
16:0	7.83 ± 0.034	7.34 ± 0.030
16:1n-7	1.92 ± 0.006	1.52 ± 0.006
18:0	2.30 ± 0.021	4.38 ± 0.059
18:1n-7	2.40 ± 0.059	2.57 ± 0.015
18:1n-9	41.25 ± 0.067	43.41 ± 0.147
18:2n-6	15.74 ± 0.036	16.56 ± 0.038
18:3n-3	6.34 ± 0.024	6.69 ± 0.016
20:1n-9	4.29 ± 0.014	3.41 ± 0.021
20:5n-3	2.47 ± 0.012	1.82 ± 0.015
22:1n-11(13)	4.57 ± 0.021	2.88 ± 0.322
22:1n-9	0.73 ± 0.005	1.00 ± 0.288
22:6n-3	2.27 ± 0.018	1.75 ± 0.035

^a^Data (mean ± SE) expressed as area percentage of identified FAME (fatty acid methyl esters) on an as-fed basis, for fatty acids present at ≥1.00% of total

Atlantic salmon smolts were transported from a local farm and held at the JBARB (Dr. Joe Brown Aquatic Research Building, Ocean Sciences Centre, St. John’s, Newfoundland, Canada) in a 3800 L tank for four months, using a flow-through seawater system. Two weeks prior to the beginning of the experiment, fish were PIT (passive integrated transponder) tagged and then randomly distributed into eight 620 L tanks (40 fish per tank and 4 replicate tanks per dietary group). Fish [initial weight (*n* = 160; mean ± SE): FO7, 178.64 ± 2.2 g; FO5, 179.28 ± 2.39 g] were fed to satiation using the experimental diets twice a day at ~12 °C and under 12-h light photoperiod for 16 weeks. Fish growth performance (i.e. fish fork length and weight) was measured at the beginning and the end of the 14 week feeding trial, and fish were held under the experimental conditions for 2 extra weeks before cell isolation and sampling; water quality parameters (e.g. temperature and oxygen saturation) were checked daily during the feeding trial. Fish growth did not significantly vary between FO7 and FO5 groups after 14 weeks of the feeding trial [final weight (mean ± SE): FO7, 340.6 ± 5.97 g (*n* = 138); FO5, 339.7 ± 6.21 g (*n* = 140)]. Fish were subjected to starvation 24 h before any handling or sampling. Fish were also anesthetized using MS222 (50 mg L^−1^; Syndel Laboratories, Vancouver, BC, Canada) before any handling procedures.

All procedures in the current study were approved by Memorial University of Newfoundland’s Institutional Animal Care Committee, according to the guidelines of the Canadian Council on Animal Care.

### Macrophage-like cell isolation

Atlantic salmon anterior (head) kidney cells were isolated as in previous studies on salmon macrophages [32–34] with some modifications. Briefly, Atlantic salmon were euthanized with an overdose of MS222 (400 mg L^−1^; Syndel Laboratories). After dissection, the head kidney was removed and transferred into Leibovitz-15+ (L-15+; Gibco, Carlsbad, CA, USA) medium supplemented with 2% fetal bovine serum (FBS; Gibco), 10 U ml^−1^ heparin (Sigma-Aldrich, St. Louis, MO, USA) and 100 U ml^−1^ penicillin and 100 μg ml^−1^ streptomycin (Gibco). Head kidney samples were then minced using 100 μm nylon cell strainers (Thermo Fisher Scientific, Waltham, MA, USA), and the resulting cell suspension was washed and pelleted by centrifugation at 400×g for 5 min at 4 °C. The cell suspension was centrifuged on a discontinuous 34/51% Percoll gradient (GE Healthcare, Uppsala, Sweden) at 400×g for 30 min in 4 °C, and the interface enriched in monocyte/macrophage-like cells was collected. The cells were washed twice (400×g for 5 min at 4 °C) and suspended in L-15+ with 2% FBS and without heparin.

The cells were counted using a hemocytometer and then seeded into 6-well plates (Corning™, Corning, NY, USA) at an equal density of 3 × 10^7^ viable cells (in 2 ml L-15+) per well. The cell viability was above 96% as determined by a trypan blue (Sigma-Aldrich) exclusion method. The cells were cultured overnight (16 h) at 15 °C, and the non-adherent cells were removed by washing the plates 3 times with L-15+. The cells were then cultured in L-15+ containing 5% FBS at 15 °C. Monocyte/macrophage-like cells are henceforth referred to as macrophage-like cells (MLCs).

### Sampling and stimulation of MLCs in dietary groups for gene expression analysis

Two fish per replicate tank in each dietary group were used for pIC stimulation and global gene expression analyses (i.e. 8 biological replicates per group). MLCs of each fish were isolated as previously described in the cell isolation section, and the cells were seeded in 6-well plates (i.e. 3 × 10^7^ cells per well). A stock solution of pIC [Sigma-Aldrich; 10 mg ml^−1^ in phosphate-buffered saline (PBS)] was prepared. Starting 24 h after seeding, MLCs isolated from each fish were exposed to PBS (control) or 10 μg ml^−1^ pIC (i.e. 1 μl of the stock solution per ml of L-15+) at 15 °C. Samples from each individual were lysed by pipetting using 800 μl of TRIzol (Invitrogen, Burlington, Ontario, Canada) at 6 (*n* = 6) and 24 (*n* = 8) h post-stimulation (HPS). Since the number of cells isolated from 2 individuals (out of 8 fish) in each dietary group was not enough for seeding 4 culture wells, the pIC- and PBS-treated cells from these individuals were only sampled at 24 HPS. The collected samples were kept at −80 °C until RNA extraction and analyses. An overview of the experimental design is illustrated in Additional file 1: Fig. S1.

Based upon a pilot study described in the last section of methods (i.e. determination of time-dependent response of salmon MLCs to pIC), 24 HPS was used as the main time point for microarray and qPCR analyses, and 6 HPS samples were collected to assess the early pIC response of a subset of microarray-identified transcripts selected for qPCR validation.

### Sampling of MLCs for cellular activity analyses

In addition, MLCs were isolated from 11 fish fed the FO7 diet and 9 fish fed the FO5 diet (from 4 tanks in FO7 and 3 tanks in FO5). We excluded one of the FO5 replicate tanks from sampling since fish in that tank were exposed to hypoxia stress after the first sampling (i.e. gene expression sampling; see the cell isolation section). The isolated cells were seeded in 6-well plates at an equal density of 10^7^ viable cells per well.

#### Phagocytosis assay

Starting 24 h after seeding, MLCs were washed once in culture medium, and 1 μm Fluoresbrite YG (yellow-green) microspheres (Polysciences, Warrington, PA) were added at a ratio of 1:30 (cell: microsphere) [35]. Twenty-four hours after microsphere exposure and culturing at 15 °C, MLCs were rinsed with culture medium and de-adhered using 500 μl of trypsin-EDTA (0.25%; Thermo Fisher Scientific, Waltham, MA). Thereafter, the trypsinized MLCs were diluted in 5 ml of culture medium, centrifuged (5 min at 500×g) at 4 °C and re-suspended in 500 μl of fluorescence-activated cell sorting (FACS) buffer (PBS + 1% FBS). Fluorescence was detected and analysed from 10,000 cells using a BD FACS Aria II flow cytometer and BD FACS Diva v7.0 software (BD Biosciences, San Jose, CA). The percentage of cells that phagocytized beads, as well as the number of beads phagocytized per cell, were determined as FITC positive events. Cell death was assessed as propidium iodide (PI) positive events, and the dead cells were excluded from analyses.

#### Respiratory burst (RB) assay

MLCs were rinsed once with culture medium and then incubated in 500 μl of respiratory burst assay buffer (L-15 media +1% BSA + 1 mM CaCl_2_) for 15 min. One microlitre of dihydrorhodamine 123 (DHR) (5 mg ml^−1^) was diluted in 1 ml of PBS and used as a stock solution; then, 50 μl of the solution were added to the cells for 15 min. DHR is a non-fluorescent dye that becomes fluorescent rhodamine under reactive oxygen conditions. Following DHR addition, 200 μM of phorbol myristate acetate (PMA), or PBS for a negative control, was added to MLCs for 45 min to stimulate reactive oxygen species (ROS) production [36]. Afterwards, MLCs were removed from the plates using trypsin-EDTA, and re-suspended in FACS buffer (PBS + 1% FBS) as described in the phagocytosis assay section. Fluorescence detection and analyses were performed using 10,000 cells, a BD FACS Aria II flow cytometer and BD FACS Diva v7.0 software (BD Biosciences). The negative control cells were used to set the baseline for non-ROS producing cells. The percentage of MLCs that produced ROS was determined as cells with rhodamine fluorescence levels greater than the negative control, and PI positive cells were excluded from analyses.

### Fatty acid analysis

HKLs were sampled from 4 replicate FO7 tanks (11 individuals) and 3 replicate FO5 tanks (10 individuals), as explained in the cell isolation section. After Percoll gradient centrifugation (see the Macrophage-like cell isolation section of methods), the interface was taken and pelleted by centrifugation at 400×g for 5 min at 4 °C. The pelleted cells were re-suspended in PBS, and washed twice in a glass tube by centrifugation at 400×g for 5 min at 4 °C. The resulting HKLs, enriched in monocyte/macrophage-like cells, were covered with 3 ml of chloroform (HPLC-grade), and the headspace of each tube was filled with nitrogen. Thereafter, the tubes were capped tightly, sealed using Teflon tape and stored at −20 °C until lipid extraction.

#### Lipid and fatty acid analyses

Lipid content of the samples was extracted based on Parrish [37]. Lipid class composition of the samples was determined using an Iatroscan Mark VI TLC–FID (Mitsubishi Kagaku Iatron, Inc., Tokyo, Japan) [38]. The fatty acid profile of the samples was measured after fatty acid methyl ester (FAME) derivatization as previously described by Hixson et al. [39]. We also used reagents and equipment similar to Hixson et al. [39] for lipid and fatty acid analyses.

The lipid class and fatty acid data were analysed using SPSS v16.0.0 (Armonk, North Castle, NY). Firstly, the normality of data was assessed using the Kolmogorov-Smirnov normality test. The differences between lipid class and fatty acid profile of HKLs of fish in different dietary groups were determined using an unpaired t-test (*p* ≤ 0.05).

### RNA extraction and purification

Total RNA was extracted from the TRIzol-lysed samples following the manufacturer’s instructions. To degrade any residual genomic DNA, total RNA samples were treated with 6.8 Kunitz units of DNase I (RNase-Free DNase Set, Qiagen, Mississauga, Ontario, Canada) with the manufacturer’s buffer (1X final concentration) at room temperature for 10 min. DNase-treated RNA samples were column-purified using the RNeasy MinElute Cleanup Kit (Qiagen) following the manufacturer’s instructions. RNA integrity was verified by 1% agarose gel electrophoresis, and RNA purity was assessed by A260/280 and A260/230 NanoDrop UV spectrophotometry. Column-purified RNA samples had A260/280 and A260/230 ratios above 1.8.

### Microarray experimental design and hybridization

MLCs, isolated from 6 individuals (i.e. samples from three replicate tanks) in each dietary group, and stimulated with pIC or PBS for 24 h, were subjected to microarray analyses [i.e. 12 samples from each dietary group (6 pIC and 6 PBS), 24 samples in total; see Additional file 1: Fig. S1]. The microarray experiment was designed and performed according to the MIAME guidelines [40]. These analyses were carried out using the consortium for Genomic Research on All Salmonids Project (cGRASP)-designed Agilent 44K salmonid oligonucleotide microarray [41] as described in Xue et al. [42]. Briefly, anti-sense amplified RNA (aRNA) was in vitro transcribed from 800 ng of each individual sample RNA (DNase-treated and column-purified) using the Amino Allyl MessageAmp™ II aRNA Amplification Kit (Ambion, Carlsbad, CA, USA) following the manufacturer’s instructions. The quality and quantity of the aRNAs were checked by agarose gel electrophoresis and NanoDrop spectrophotometry. Amplified RNA from all 24 samples (i.e. 10 μg from each sample) was pooled and used as a common reference in this experiment. Twenty micrograms of aRNA (i.e. experimental samples or common reference) were precipitated, using standard ethanol precipitation methodology, and re-suspended in coupling buffer. Thereafter, the experimental samples were labelled with Cy5 (GE Healthcare Life Sciences, Buckinghamshire, UK), and the common reference was labelled with Cy3 (GE Healthcare Life Sciences), following the manufacturer’s instructions. The efficiency of labelling and aRNA concentration were assessed using spectrophotometry (i.e. microarray feature in NanoDrop). The labeled aRNA (i.e. 825 ng) from each experimental sample was mixed with an equal amount of labelled aRNA from the common reference, and the resulting pool was fragmented following the manufacturer’s instructions (Agilent, Mississauga, ON). Each labelled aRNA pool (i.e. an individual sample and common reference) was co-hybridized to a 44K microarray at 65 °C for 17 h with rotation (10 rpm) using an Agilent hybridization oven.

### Microarray data acquisition and analysis

The microarray slides were scanned at 5 μm resolution with 90% of laser power using a ScanArray Gx Plus scanner and ScanExpress v4.0 software (Perkin Elmer, Waltham, Massachusetts, USA), and the Cy3 and Cy5 channel photomultiplier tube (PMT) settings were adjusted to balance the fluorescence signal. The raw data were saved as TIFF images, and the signal intensity data were extracted using Imagene 9.0 (BioDiscovery, El Segundo, California, USA). Using R and the Bioconductor package marray, the low-quality or flagged spots on the microarray were discarded from datasets, followed by log_2_-transformation and Loess-normalization of data [19]. Thereafter, probes with absent values in more than 25% of all 24 arrays were omitted from the dataset, and the missing values were imputed using the EM_array method and the LSimpute package [19, 43, 44]. The final dataset that was used for statistical analyses consisted of 12,983 probes for all arrays (GEO accession number: GSE93773).

The differentially expressed probes (DEP) between different treatments were determined using Significance Analysis of Microarrays (SAM) [45] and Rank Products (RP) [46, 47]. We used the Excel add-in SAM package (Stanford University, CA) and two-class comparison analysis with a false discovery rate (FDR) cutoff of 0.05 to identify the diet-responsive transcripts between groups (i.e. FO7, PBS vs. FO5, PBS; and FO7, pIC vs. FO5, pIC) and pIC-responsive transcripts within groups (e.g. FO7, PBS vs. FO7, pIC). The diet- and pIC-responsive transcripts were also found using RP analysis at a percentage of false-positives (PFP) threshold of 0.05, as implemented by the Bioconductor package. The resulting significant transcript lists were re-annotated using contigs or singletons [41] that were used for designing the given informative 60mer oligonucleotide probes on the array.

The BLASTx searches of NCBI’s non-redundant (nr) amino acid sequence database (E-value <1e-05) were carried out using Blast2GO software (BioBam Bioinformatics S.L., Valencia, Spain) [48, 49]. The resulting BLASTx hits were mapped to gene ontology (GO) terms of pIC-responsive transcripts in each dietary group (GO Biological Process level 2). GO enrichment analysis was performed (Fisher’s exact test, FDR cutoff of 0.05) using Blast2GO software. The Ancestor Chart feature of QuickGO (http://www.ebi.ac.uk/QuickGO) was used to categorise and select a subset of enriched GO terms related to immunity. We used the Pearson correlation and complete linkage clustering function in the Genesis software (Rockville, Maryland, USA) [50] for the hierarchical clustering of median-centred data of DEP as described in Booman et al. [19].

### qPCR validation

Transcript levels of a subset of genes identified as differentially expressed in the microarray analyses were validated using qPCR. These genes included a subset of diet-responsive up- or down-regulated transcripts identified by RP analysis. Additionally, pIC-responsive transcripts (e.g. up- and down-regulated) that are involved in different molecular functions (e.g. pathogen recognition, signal transduction, transcription factors and immune effectors) and immune pathways [e.g. IFN (interferon) and TLR] were selected for qPCR validation (Additional file 2: Table S1). These transcripts were mainly selected from pIC-responsive transcripts in both dietary groups, and overlapping between the SAM and RP analyses. We assessed the expression of two transcripts (*tlr3* and *tlr7*) that play important roles in dsRNA signalling pathways but were absent from the microarray platform. In addition, *mx-b* was included in the qPCR analyses since this showed a dietary rapeseed-dependent expression in head kidney of pIC-stimulated salmon in our previous study [18]. Transcript levels of these genes of interest (GOIs) were measured in all of the samples (i.e. both PBS- and pIC-treated) from each dietary group collected at both 6 and 24 HPS.

First-strand cDNA templates for qPCR were synthesized in 20 μl reactions from 800 ng of DNaseI-treated, column-purified total RNA using random primers (250 ng; Invitrogen) and M-MLV reverse transcriptase (200 U; Invitrogen) with the manufacturer’s first-strand buffer (1X final concentration) and DTT (10 mM final concentration) at 37 °C for 50 min.

All PCR amplifications were performed in 13 μl reactions using 1X Power SYBR Green PCR Master Mix (Applied Biosystems, Burlington, Ontario, Canada), 50 nM of both the forward and reverse primers, and the indicated cDNA quantity (see below). Amplifications were performed using the ViiA 7 Real-Time PCR system (384-well format) (Applied Biosystems); the real-time analysis program consisted of 1 cycle of 50°C for 2 min, 1 cycle of 95 °C for 10 min and 40 cycles of 95 °C for 15 s and 60 °C for 1 min, with fluorescence detection at the end of each 60 °C step.

The qPCR assays used in the current study were designed and performed following MIQE guidelines [51]. Primers used in this study were designed using Primer3web v4.0.0 (http://primer3.ut.ee/) (Additional file 2: Table S1). The performance and amplification efficiencies of all primer pairs were tested prior to use in the experimental studies. Briefly, for diet-responsive transcripts, they were assessed using a cDNA template generated from a pool of 8 individuals from pIC- and PBS-stimulated samples at 24 HPS from both dietary groups; for pIC-responsive up-regulated transcripts, they were assessed using a cDNA template generated from a pool of 6 individuals from pIC-stimulated samples at 24 HPS from both dietary groups; for pIC-responsive down-regulated transcripts, they were assessed using a cDNA template generated from a pool of 6 individuals from PBS-stimulated samples at 24 HPS from both dietary groups. The standard curves for all primer pairs (i.e. GOIs and candidate normalizers) were generated using a 5-point, 3-fold serial dilution of the given cDNA template (starting with cDNA representing 10 ng of input total RNA) as well as a no-template control. The primer quality tests were performed in triplicate. Only primer pairs generating an amplicon with a single melting peak, no primer-dimer present in the no-template control, and an acceptable amplification efficiency (i.e. 80–110%) [52] were used for qPCR analyses (Additional file 2: Table S1).

Transcript levels of the GOIs were normalized to transcript levels of two endogenous control genes. To select these endogenous controls, qPCR primers pairs were designed for seven candidate normalizers, [i.e. *actb* (*beta-actin*), *rpl32* (*60S ribosomal protein 32*), *ef1a1* (*elongation factor 1 alpha-1*), *pabpc1* (*polyadenylate-binding protein cytoplasmic 1*), *eif3d* (*eukaryotic translation initiation factor 3 subunit D*), *tubg1* (*tubulin gamma-1*) and *ntf2* (*nuclear transport factor 2*)], and quality tested as described above. Thereafter, the fluorescence threshold cycle (C_T_) values of 50% of the experimental samples (including PBS- and pIC-treated samples at both 6 and 24 HPS from both dietary groups) were measured in duplicate for each of these transcripts using cDNA representing 3.2 ng of input total RNA, and then analysed using geNorm in the qBase software [53]. Two transcripts, *eif3d* and *rpl32*, were expressed comparably (i.e. with the lowest M-values; measure of transcript expression stability) in all samples tested and thus were selected as the normalizers for the experimental qPCR assays.

When primer quality testing and normalizer selection were completed, qPCR analyses of transcript (mRNA) expression levels of the GOIs were performed. In all cases, cDNA representing 3.2 ng of input RNA was used as template in the PCR reactions. On each plate, for every sample, the GOIs and endogenous controls were tested in triplicate, and a plate linker sample (i.e. a sample that was run on all plates in a given study) and a no-template control were included. The relative quantity (RQ) of each transcript was determined using the ViiA 7 Software Relative Quantification Study Application (Version 1.2.3) (Applied Biosystems), with normalization to both *eif3d* and *rpl32* transcript levels, and with amplification efficiencies incorporated. For each GOI, the sample with the lowest normalized expression (mRNA) level was set as the calibrator sample (i.e. assigned an RQ value = 1).

RQ values of each transcript of interest were subjected to statistical analyses. Prior to analyses, the normality of data was checked using the Kolmogorov-Smirnov normality test. A two-way ANOVA test was applied to analyse qPCR results between dietary groups (e.g. FO7, PBS vs. FO5, PBS), whereas the significant differences within each dietary group (between pIC and PBS) were assessed using a repeated measures two-way ANOVA test. These analyses were followed by Sidak multiple comparison post hoc tests to determine significant differences (*p* ≤ 0.05) in the time- and treatment-matched results between dietary groups as well as the significant differences in time-matched pIC or PBS groups within each dietary group and within pIC and PBS groups at different time points. All data analyses of qPCR results in the current study were conducted in the Prism package v6.0 (GraphPad Software Inc., La Jolla, CA, USA).

### Determination of time-dependent response of salmon MLCs to pIC

Prior to the diet-related experiment and to determine the time-dependent response to pIC, salmon MLCs were isolated from 4 individuals, weighing 1.78 ± 0.09 kg, as described in the cell isolation section. The resulting cells were seeded into 35 mm (i.e. similar size to one well of a 6-well plate) culture dishes (Corning™) at an equal density of 3 × 10^7^ viable cells per dish. MLCs from each individual were incorporated into all groups and sampling points. After 24 h of culture, MLCs were treated with PBS or 10 μg ml^−1^ pIC (Sigma-Aldrich) (stimulative dose of pIC for salmon MLCs [54]); then, the samples were collected at 3, 6, 12, 24 and 48 HPS by removing the medium and adding 800 μl of TRIzol (Invitrogen). RNAs were extracted as described previously. The expression of selected biomarker genes [i.e. *gig1*, *mx*, *viperin* and *lgp2* (*RNA helicase lgp2*)] involved in the antiviral immune response was assessed by qPCR (see the qPCR validation section).

Expression levels of all of the assayed antiviral biomarker transcripts were significantly up-regulated by pIC at 12 HPS, peaked at 24 HPS and were significantly lower within the pIC group at 48 HPS compared to 24 HPS (data not shown). Since the peak of pIC response in salmon macrophages occurred at 24 HPS, this time point was chosen for the global gene expression analyses of pIC-stimulated MLCs in the diet-related experiment. Additionally, there were non-significant increases in expression of *gig1*, *mx* and *viperin* and a significant up-regulation of *lgp2* in response to pIC at 6 HPS (data not shown); therefore, since the early pIC response in salmon MLCs occurred at 6 HPS, this time point was included in the qPCR studies.

## Results

### Phagocytosis and RB

**Fig. 1:**
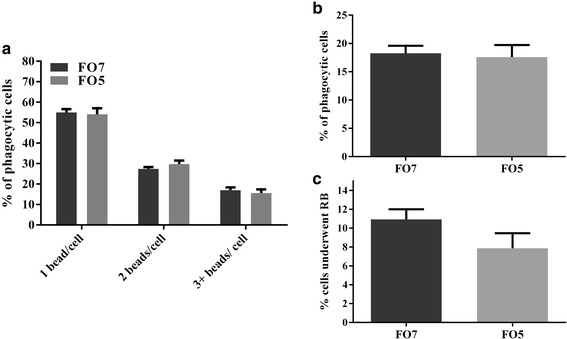
Cellular functions of macrophage-like cells (MLCs) isolated from salmon in FO7 and FO5 dietary groups. Data are presented as mean ± SE. ﻿No significant differences (*p* > 0.05) were found between groups, using an unpaired t-test. **a** phagocytosis of MLCs based on the number of the beads ingested by phagocytic cells, **b** The percentage of phagocytic salmon MLCs in dietary groups, **c** The percentage of salmon MLCs in each dietary group that underwent respiratory burst (RB)

In this study, the phagocytosis and RB of the salmon MLCs were not significantly influenced by diet (Fig. 1).

### Lipid and fatty acid analyses

There were some differences in the composition of lipid classes in HKLs isolated from salmon fed different levels of dietary vegetable oil (Table 2). The proportion of free fatty acids of HKLs was significantly higher in the FO5 group (1.99 ± 0.44%) than in the FO7 group (0.58 ± 0.16%) (Table 2). There was a significant increase in sterols of the cells isolated from salmon on FO5 diet compared to those on FO7 diet (Table 2). However, HKLs of salmon in the FO5 group had lower phospholipid proportions compared to the FO7 group. The phospholipids were found to be the most dominant lipid class in salmon HKLs. The between-group variations in other lipid classes (i.e. hydrocarbons and triacylglycerols) of salmon in this experiment were not statistically significant (Table 2).

**Table 2:** Lipid class and fatty acid composition of salmon head kidney leukocytes (HKLs) in different dietary groups

Lipid class %	FO7	FO5	*p* value
Hydrocarbons	0.76 ± 0.104	0.43 ± 0.177	0.110
Triacylglycerols	0.83 ± 0.182	1.94 ± 0.641	0.124
Free fatty acids	0.58 ± 0.162	1.99 ± 0.442	**0.012**
Sterols	11.38 ± 0.318	12.51 ± 0.371	**0.032**
AMPL^a^	5.71 ± 0.787	6.06 ± 0.909	0.777
Phospholipids	80.74 ± 0.816	76.80 ± 1.695	**0.044**
Fatty acids %^b^			
14:0	1.17 ± 0.027	0.97 ± 0.030	**< 0.0001**
15:0	0.24 ± 0.003	0.22 ± 0.005	**0.001**
16:0	18.28 ± 0.189	17.96 ± 0.245	0.314
16:1n-7	0.77 ± 0.032	0.73 ± 0.050	0.473
17:0	0.21 ± 0.004	0.21 ± 0.005	0.200
16:4n-1	2.19 ± 0.289	2.08 ± 0.356	0.814
18:0	6.36 ± 0.110	6.45 ± 0.149	0.621
18:1n-9	17.08 ± 0.337	17.81 ± 0.349	0.151
18:1n-7	3.15 ± 0.031	3.16 ± 0.043	0.840
18:2n-6	4.58 ± 0.084	4.93 ± 0.142	**0.041**
18:3n-6	0.20 ± 0.005	0.27 ± 0.011	**< 0.0001**
18:3n-3	0.76 ± 0.025	0.79 ± 0.038	0.568
18:4n-3	0.26 ± 0.008	0.30 ± 0.023	0.073
20:1n-9	1.14 ± 0.037	0.98 ± 0.036	**0.005**
20:2n-6	0.63 ± 0.020	0.63 ± 0.030	0.922
20:3n-6	1.57 ± 0.046	1.88 ± 0.078	**0.003**
20:4n-6	5.23 ± 0.152	5.67 ± 0.190	0.080
20:4n-3	0.56 ± 0.017	0.55 ± 0.018	0.739
20:5n-3	6.37 ± 0.150	5.77 ± 0.131	**0.008**
22:1n-11(13)	0.24 ± 0.024	0.32 ± 0.062	0.232
22:1n-9	0.27 ± 0.051	0.46 ± 0.112	0.131
22:5n-3	0.74 ± 0.027	0.69 ± 0.023	0.245
22:6n-3	23.74 ± 0.448	23.00 ± 0.473	0.271
24:1	0.72 ± 0.024	0.74 ± 0.029	0.450
Bacterial	1.14 ± 0.047	1.05 ± 0.049	0.196
Σ SFA^c^	26.41 ± 0.298	25.94 ± 0.399	0.346
Σ MUFA^d^	24.48 ± 0.397	25.25 ± 0.482	0.229
Σ PUFA^e^	48.53 ± 0.594	48.28 ± 0.624	0.774
Σ LC n-3^f^	31.58 ± 0.551	30.16 ± 0.531	0.080
Σ LC n-6	7.68 ± 0.171	8.47 ± 0.226	**0.012**
LCn-6/ LCn-3	0.24 ± 0.005	0.28 ± 0.007	**0.001**
P/S	1.84 ± 0.040	1.87 ± 0.047	0.662
Σ n-3	33.43 ± 0.583	32.13 ± 0.449	0.098
DHA/EPA ratio	3.74 ± 0.087	4.00 ± 0.102	0.068

Values are mean ± SE﻿. Bold *p* values indicate a significant (*p* < 0.05) difference between groups

^a^Acetone mobile polar lipids

^b^Data are expressed as area percentage of identified FAME (fatty acid methyl ester), for fatty acids that were present at ≥0.2% of the total

^c^Saturated fatty acid

^d^Monounsaturated fatty acid

^e^Polyunsaturated fatty acid

^f^Long chain n-3

Other fatty acids present at <0.2%: 15:0, *i*16:0, 16:1n-5, *i*17:0, *ai*17:0, 16:2n-4, 17:1, 18:2n-4, 18:3n-4, 20:0, 20:3n-3, 22:0, 22:5n-6

HKLs isolated from salmon in both dietary groups showed a comparable profile for many fatty acids (Table 2). However, significant changes were found in some fatty acids between the two groups. For example, linoleic acid (18:2n-6) and dihomo-gamma-linolenic acid (20:3n-6) were higher in HKLs isolated from fish in the FO5 group than those of FO7. Nonetheless, EPA (20:5n-3) of salmon HKLs decreased in the FO5 group compared to FO7 group (Table 2). The sum of long-chain n-6 fatty acids (LCn-6) and the LCn-6/LCn-3 ratio of HKLs significantly increased in the FO5 group compared to the FO7 group.

### Microarray results

#### The diet-responsive transcripts in salmon MLCs

**Fig. 2:**
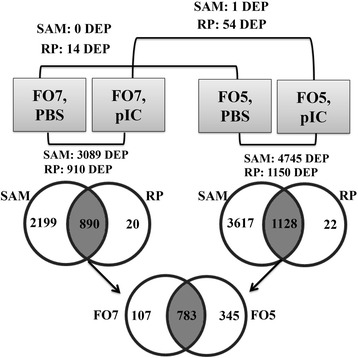
Overview of microarray results. The diet- and pIC-responsive probes identified by SAM (FDR < 0.05) and RP (PFP < 0.05) analyses. The differentially expres﻿sed probes (DEP) by diet and pIC are shown in Additional file 3: Table S2 and Additional file 4: Table S3, respectively

To identify diet- and pIC-responsive transcripts in salmon MLCs, we analysed the expression data using both SAM and RP. Only one DEP was found by SAM between the two diets (FO5/FO7) in pIC-stimulated samples (i.e. *transmembrane protein 115 ­like*; 1.87-fold up-regulated in FO5). RP identified 14 and 54 DEP (PFP < 0.05) between the two diets in the PBS and pIC groups, respectively (Fig. 2). However, most (12 out of 14) of the diet-responsive probes between the PBS treatments were also differentially expressed between the pIC groups of the two diets, and they showed a similar expression trend (i.e. up- or down-regulation response to a given diet) in both comparisons. Additional file 3: Table S2 shows the diet-responsive probes in the pIC and PBS groups. Transcripts involved in lipid metabolism (e.g. *fatty acid-binding protein, adipocyte*; *fabp4*) as well as immune responses (e.g. *Fc receptor-like protein* *2 * and *MHC-I*) were identified as DEP by RP. A subset of 9 diet-responsive transcripts was subjected to qPCR validation.

#### The pIC-responsive transcripts in salmon MLCs

Additional file 4 Table S3 presents the pIC-responsive probes within FO5 or FO7 groups. SAM showed 3089 DEP (FDR < 0.05) by pIC within the FO7 group (pIC vs. PBS), whereas RP identified 910 DEP (PFP < 0.05) within this group (3109 DEP in total). Also, SAM found 4745 DEP (FDR < 0.05) by pIC within the FO5 group (pIC vs. PBS), but RP identified 1150 DEP (PFP < 0.05) in this group (4767 DEP in total). Venn diagrams showed that 890 and 1128 DEP overlapped between the SAM and RP significant pIC-responsive gene lists of FO7 and FO5 groups, respectively. Between these pIC-responsive probes, 107 and 345 of them were only identified as SAM- and RP-overlapped in the FO7 and FO5 groups, respectively. Figure 2 illustrates the microarray results and overlapping pIC-responsive probes between experimental groups and analyses methods. SAM and RP apply distinct approaches to detect DEP in microarray experiments [45, 46], and the overlap of transcripts identified by both techniques represent very high-trust gene lists (i.e. few false positives) as demonstrated by Brown et al. [47]. Therefore, the microarray-identified pIC-responsive probes that overlapped between the SAM and RP analyses in each group were subjected to further functional analyses (i.e. GO analysis and Fisher’s exact test)

#### Hierarchical clustering analyses of microarray results

**Fig. 3:**
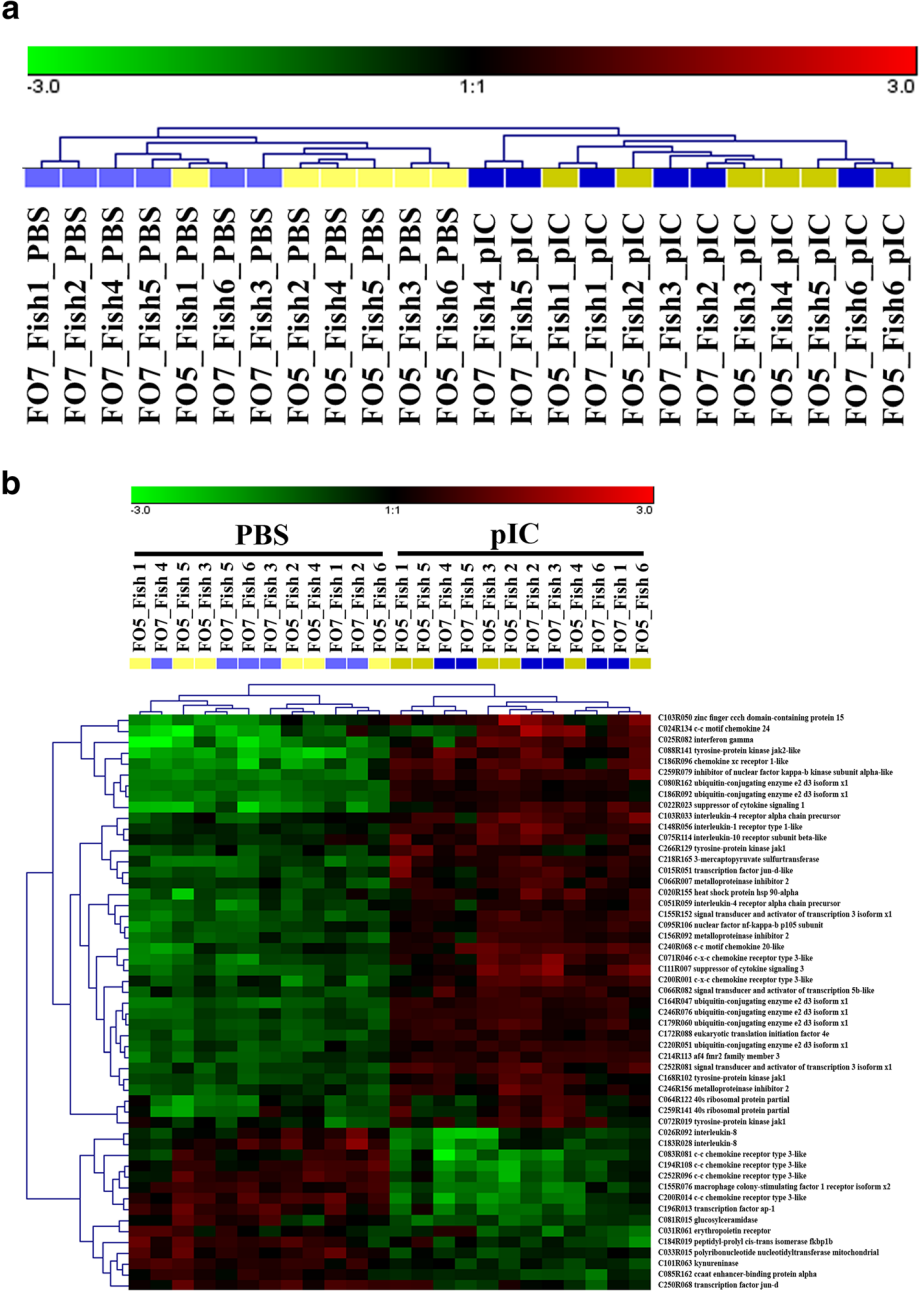
Hierarchical clustering analyses; **a** Clustering of samples using the whole microarray dataset; **b** Clustering of samples based on a subset of pIC-responsive transcripts involved in Cytokine-mediated pathway [i.e. associated with GO terms “cellular response to cytokine stimulus” (GO:0071345) and/or “response to cytokine” (GO:0034097)]; the transcript names are derived from the significant BLASTx hits (E-value <1e-05) as implemented by Blast2GO. Coloured blocks at the top of the figures indicate the dietary and stimulation groups: light blue, PBS FO7; dark blue, pIC FO7; light yellow, PBS FO5; dark yellow, pIC FO5

Hierarchical clustering analyses were performed to determine if samples isolated from a dietary group shared a similar transcriptome profile. We used the whole microarray dataset (Fig. 3a), the pIC-responsive probes (i.e. 1235 DEP) identified by both SAM and RP (Additional file 5: Fig. S2) and a subset of pIC-responsive transcripts involved in response to cytokine (i.e. transcripts associated with cellular response to cytokine stimulus and/or response to cytokine GO terms; Fig. 3b) for hierarchical clustering analyses. Samples from pIC or PBS treatments of both dietary groups showed similar transcriptome profiles (i.e. whole microarray dataset), as they were only clustered together according to the stimulation groups (Fig. 3a). The majority of the samples in PBS treatment of each diet (i.e. 5 samples in FO5 and 4 samples in FO7) grouped closely together (Fig. 3a), indicating similar constitutive global gene expression of MLCs isolated from a given diet; however, this diet-related clustering was not found in pIC-stimulated samples (Fig. 3a). Using a subset of the pIC-responsive probes (Additional file 5: Fig. S2), the samples were separated into two clusters based upon their stimulation group (i.e. pIC and PBS), and no grouping was detected based on the dietary treatment. Similar results were observed for clustering of samples using a subset of 53 pIC-responsive probes with putative roles in response to cytokines [i.e. transcripts associated with cellular response to cytokine stimulus (GO:0071345) and/or response to cytokine (GO:0034097) GO terms] (Fig. 3b).

#### GO terms and GO enrichment analyses of pIC-responsive transcripts in dietary groups

GO terms (i.e. Molecular Function, Biological Process, or Cellular Component categories) of DEP by pIC treatment in each dietary group were obtained (see Additional file 4: Table S3). The GO annotation distributions (Biological Process level 2) of pIC-responsive transcripts overlapping between SAM and RP analyses in each dietary group were created (Additional file 6: Fig. S3). The proportions of pIC-responsive transcripts associated with different GO annotation (e.g. signalling and immune system process) in the FO7 group were highly comparable to those of the FO5 group.

The Fisher’s exact test (FDR < 0.05) was used to determine the over- and under-represented GO terms of the pIC-responsive transcripts (i.e. overlapped between SAM and RP) in each dietary group compared to the whole array. This analysis showed 110 and 117 significantly enriched GO terms by pIC stimulation in the FO7 and FO5 groups, respectively (see Additional file 7: Table S4). Moreover, 88 enriched GO terms by pIC treatment overlapped in both lists and they shared a similar trend (i.e. over- or under-represented GO terms) between two dietary groups (see Additional file 7: Table S4 and Table 3). Some GO terms (e.g. intracellular organelle and cytoskeleton) in the Cellular Component category were significantly under-represented in both groups. A subset of enriched GO terms that were associated with immune responses is presented in Table 3. GO terms involved in immune responses [e.g. cytokine receptor activity, chemokine receptor activity, response to cytokine, chemokine-mediated signalling pathway and MyD88 (myeloid differentiation primary-response gene 88)-independent Toll-like receptor signalling pathway] were significantly over-represented in pIC-responsive gene lists of both dietary groups (Table 3). Nonetheless, GO terms associated with Toll-like receptor 3 signalling pathway and negative regulation of type I interferon production were only significantly over-represented in the pIC-responsive transcript list of the FO7 group (see Table 3), and this may be influenced by the lower number of pIC-responsive probes identified in FO7 compared to that in FO5.

**Table 3:** An immune-related subset of enriched GO terms of pIC-responsive transcripts (overlap between SAM and RP analyses) within each dietary group

GO ID	GO Term^a^	Category^b^	Number of probes with GO^c^		Over/Under
			Test FO7	Test FO5	
GO:0004950	chemokine receptor activity	F	**8**	**8**	OVER
GO:0004896	cytokine receptor activity	F	**11**	**14**	OVER
GO:0071345	cellular response to cytokine stimulus	P	**37**	**43**	OVER
GO:0070098	chemokine-mediated signaling pathway	P	**9**	**9**	OVER
GO:0034097	response to cytokine	P	**40**	**50**	OVER
GO:0045647	negative regulation of erythrocyte differentiation	P	**5**	**5**	OVER
GO:0030219	megakaryocyte differentiation	P	**9**	**9**	OVER
GO:0035666	TRIF-dependent toll-like receptor signaling pathway	P	**8**	**8**	OVER
GO:0002756	MyD88-independent toll-like receptor signaling pathway	P	**8**	**8**	OVER
GO:0006954	inflammatory response	P	**25**	**29**	OVER
GO:0034138	toll-like receptor 3 signaling pathway	P	**8**	8	OVER
GO:0002250	adaptive immune response	P	**15**	16	OVER
GO:0019221	cytokine-mediated signaling pathway	P	**24**	27	OVER
GO:0032480	negative regulation of type I interferon production	P	**6**	6	OVER

^a^This subset of enriched GO terms associated with immune responses was selected using Ancestor Chart feature of the QuickGO website (http://www.ebi.ac.uk/QuickGO). The full list of enriched GO terms is presented in Additional file 7: Table S4

^b^F: Molecular Function and P: Biological Process

^c^Numbers of probes annotated with each GO term in pIC-responsive gene list overlapping between SAM and RP of each dietary group. Bold numbers indicate a significant over-representation (Fisher’s exact test, FDR < 0.05) in the pIC-responsive gene list of the given dietary treatment, compared to the whole 44K salmon microarray. Total number of probes annotated with at least 1 GO term was 666 and 865 for FO7 and FO5 groups, respectively

### qPCR validation

#### Diet-responsive transcripts

**Fig. 4:**
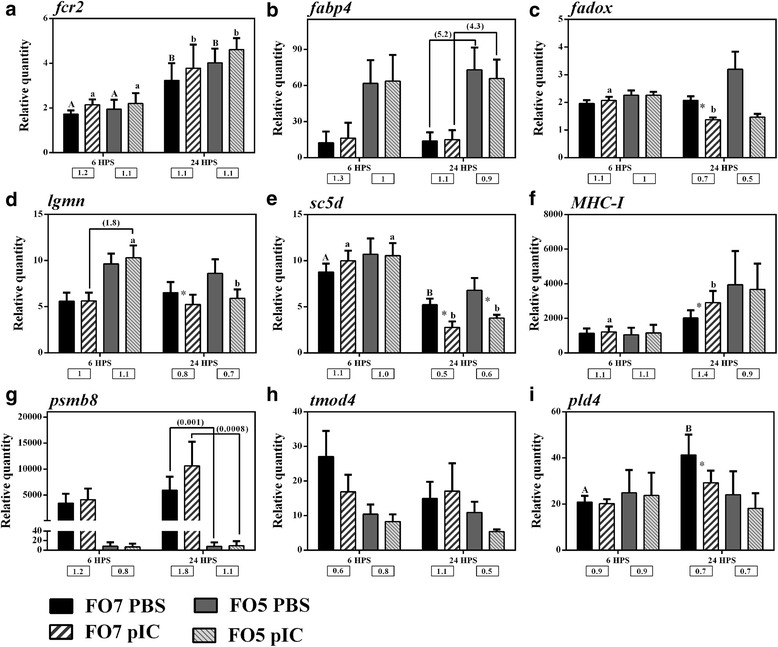
qPCR for transcripts identified by microarray as diet-responsive. Data are presented as mean ± SE. Fold changes on the line connecting the stimulation-matched treatments of two diets show the significant differences between PBS or pIC of dietary groups (*p* < 0.05). An asterisk represents significant difference between time-matched pIC and PBS groups in each dietary treatment (*p* < 0.05). Different letters (upper-case for PBS and lower-case for pIC) indicate the significant differences within PBS or pIC group of a dietary treatment over time (*p* < 0.05). The fold-change (pIC/control) values are shown below the figures. The presented legend describes the dietary (i.e. FO7 and FO5) and treatment (i.e. PBS and pIC) groups of all panels. **a**) *fcr2*; **b**) *fabp4*; **c**) *fadox*; **d**) *lgmn*; **e**) *sc5d*; **f**) *MHC-I*; **g**) *psmb8*; **h**) *tmod4*; **i**) *pld4*

A subset of 9 diet-responsive transcripts identified by RP analysis was subjected to qPCR validation (Fig. 4). Table 4 represents the comparison between microarray and qPCR results for these 9 transcripts. All of the qPCR-assayed diet-responsive transcripts, except for *MHC-I*, showed similar fold-change directions (up- or down-regulation) to microarray results (Table 4). The microarray results were significantly validated for 2 (i.e. significant differential expression) of the studied transcripts. The expression of *fabp4* significantly increased in both the PBS and pIC groups (5.2- and 4.3-fold, respectively) of the FO5 diet at 24 HPS, compared to those of the FO7 diet (Fig. 4b). On the contrary, *psmb8* (*proteasome subunit beta type-8*) expression was strongly suppressed by the FO5 diet in PBS- and pIC-stimulated salmon MLCs at 24 HPS, and the level of this transcript was very low or undetectable by the qPCR assays in the majority of the samples in the FO5 group (Fig. 4g). The RP result for *lgmn* (*legumain-like*) was not confirmed at 24 HPS, but an up-regulation similar to the microarray results was seen at 6 HPS in the pIC-treated MLCs of salmon fed FO5 diet (1.83-fold increase) compared to those fed FO7 diet (Fig. 4d and Table 4). Also, *sc5d* (*lathosterol oxidase*) and *pld4* (*phospholipase d4*) expression did not vary between the dietary treatments, but these transcripts were shown by both microarray and qPCR to be down-regulated (in at least one of the dietary groups) by pIC stimulation at 24 HPS (Fig. 4e and i; Additional file 4: Table S3).

**Table 4:** Comparison between the microarray and qPCR results of a subset of 9 diet-responsive transcripts identified by Rank Product (RP)

Microarray Probe ID	Name	FO5/FO7, Microarray fold-change PBS@ 24 HPS^1^	FO5/FO7, Microarray fold-change pIC @ 24 HPS^1^	FO5/FO7, qPCR fold-changePBS @ 6 HPS	FO5/FO7, qPCR fold-change pIC @ 6 HPS	FO5/FO7, qPCR fold-change PBS @ 24 HPS	FO5/FO7, qPCR fold-change pIC @ 24 HPS	qPCR, *p* value PBS^2^	qPCR, *p* value pIC^2^
C148R063	*Fc receptor-like protein 2* (*fcr2*)	3.63	3.72	1.13	1.03	1.24	1.21	0.42	0.53
C108R146	*fatty acid-binding protein, adipocyte (fabp4)*	2.72	2.98	4.99	3.91	5.29*	4.39*	0.0012	0.003
C126R012	*FAD-linked sulfhydryl oxidase ALR-like (fadox)*	2.21	–	1.15	1.09	1.54	1.07	0.082	0.22
C001R074	*lathosterol oxidase (sc5d)*	–	1.96	1.22	1.06	1.30	1.36	0.15	0.36
C146R053	*legumain (lgmn)*	–	1.83	1.72	1.83*	1.32	1.13	0.024^3^	0.022
C096R058	*tropomodulin-4-like (tmod4)*	–	0.43	0.38	0.49	0.73	0.31	0.039^3^	0.064
C153R016	*phospholipase d4 (pld4)*	0.43	0.47	1.19	1.17	0.58	0.62	0.46	0.56
C027R162	*MHC class I antigen*	0.29	0.32	0.92	0.95	1.95	1.26	0.44	0.72
C164R003	*proteasome subunit beta type-8 (psmb8)*	0.28	0.23	0.0024	0.0016	0.0013*	0.00088*	0.011	0.016

^1^The fold changes between PBS- or pIC-matched groups of FO5 and FO7 at the same time (FO5/FO7). A dash (−) represents no differential expression between groups for a given comparison in microarray analyses

^2^The *p* values of qPCR results as implemented by two-way ANOVA between PBS- and pIC-matched groups of dietary treatments

^3^The significant *p* values were observed for PBS-matched groups of dietary treatments, but no significant difference was found by Sidak multiple comparisons post hoc test

*Significant difference (*p* < 0.05) between pIC- and PBS-matched groups of dietary treatments in qPCR assay

#### pIC-responsive transcripts

**Fig. 5:**
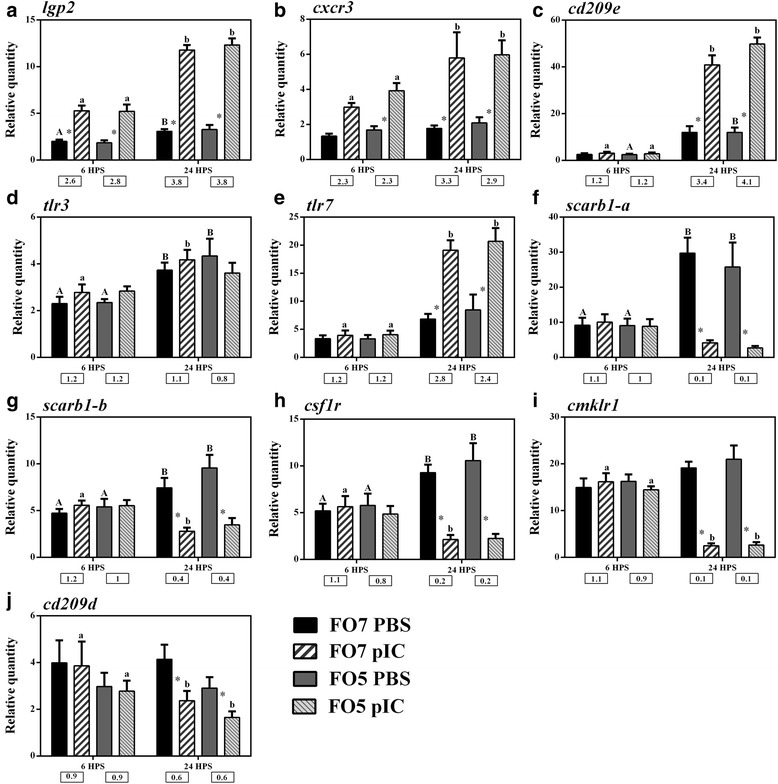
qPCR for pIC-responsive transcripts playing roles as PRRs or other receptors. Data are presented as mean ± SE. An asterisk represents significant difference between time-matched pIC and PBS groups in each dietary treatment (*p* < 0.05). Different letters (upper-case for PBS and lower-case for pIC) indicate the significant differences within PBS or pIC group of a dietary treatment over time (*p* < 0.05). The fold-change (pIC/control) values are shown below the figures. The presented legend describes the dietary (i.e. FO7 and FO5) and treatment (i.e. PBS and pIC) groups of all panels. **a**) *lgp2*; **b**) *cxcr3*; **c**) *cd209e*; **d**) *tlr3*; **e**) *tlr7*; **f**) *scarb1-a*; **g**) *scarb1-b*; **h**) *csf1r*; **i**) *cmklr1*; **j**) *cd209d*

**Fig. 6:**
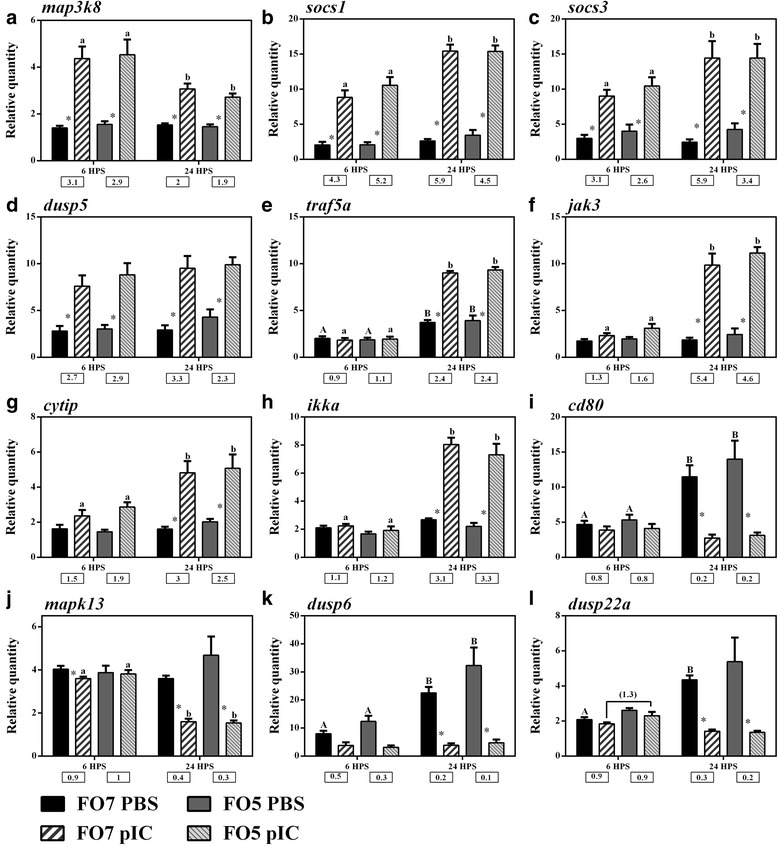
qPCR for pIC-responsive transcripts involved in signal transduction. Data are presented as mean ± SE. Fold changes on the line connecting the stimulation-matched treatments of two diets show the significant differences between PBS or pIC of dietary groups (*p* < 0.05). An asterisk represents significant difference between time-matched pIC and PBS groups in each dietary treatment (*p* < 0.05). Different letters (upper-case for PBS and lower-case for pIC) indicate the significant differences within PBS or pIC group of a dietary treatment over time (*p* < 0.05). The fold-change (pIC/control) values are shown below the figures. The presented legend describes the dietary (i.e. FO7 and FO5) and treatment (i.e. PBS and pIC) groups of all panels. **a**) *map3k8*; **b**) *socs1*; **c**) *socs3*; **d**) *dusp5*; **e**) *traf5a*; **f**) *jak3*; **g**) *cytip*; **h**) *ikka*; **i**) *cd80*; **j**) *mapk13*; **k**) *dusp6*; **l**) *dusp22a*

**Fig. 7:**
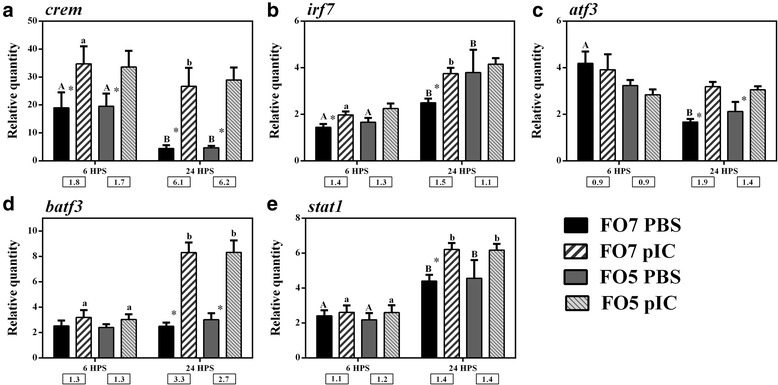
qPCR for pIC-responsive transcripts playing roles as transcription factors. Data are presented as mean ± SE. An asterisk represents significant difference between time-matched pIC and PBS groups in each dietary treatment (*p* < 0.05). Different letters (upper-case for PBS and lower-case for pIC) indicate the significant differences within PBS or pIC group of a dietary treatment over time (*p* < 0.05). The fold-change (pIC/control) values are shown below the figures. The presented legend describes the dietary (i.e. FO7 and FO5) and treatment (i.e. PBS and pIC) groups of all panels. **a**) *crem*; **b**) *irf7*; **c**) *atf3*; **d**) *batf3*; **e**) *stat1*

**Fig. 8:**
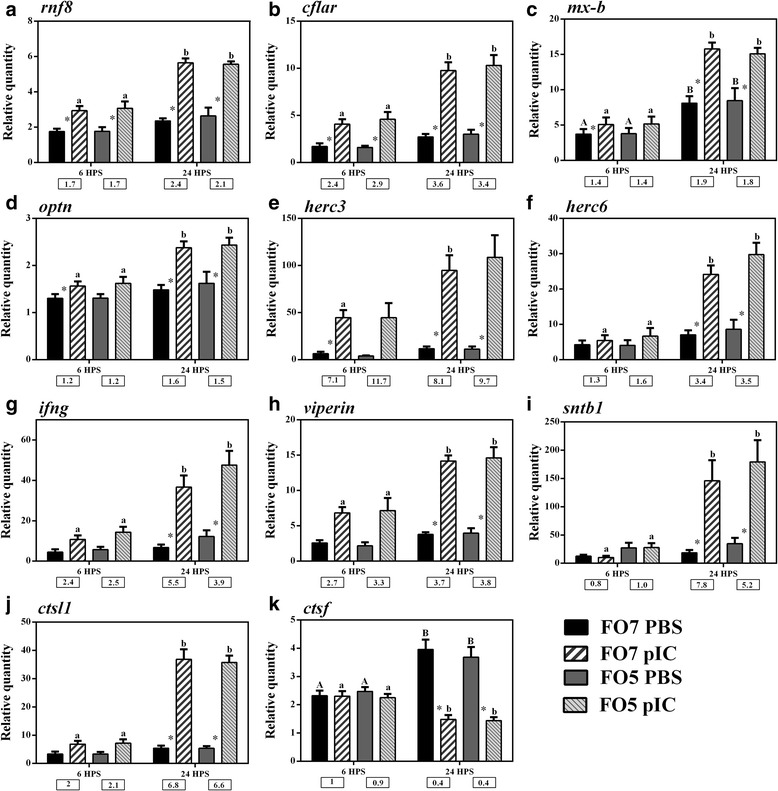
qPCR for pIC-responsive transcripts playing roles as immune effectors. Data are presented as mean ± SE. An asterisk represents significant difference between time-matched pIC and PBS groups in each dietary treatment (*p* < 0.05). Different letters (upper-case for PBS and lower-case for pIC) indicate the significant differences within PBS or pIC group of a dietary treatment over time (*p* < 0.05). The fold-change (pIC/control) values are shown below the figures. ﻿The presented legend describes the dietary (i.e. FO7 and FO5) and treatment (i.e. PBS and pIC) groups of all panels. **a**) *rnf8*; **b**) *cflar*; **c**) *mx-b*; **d**) *optn*; **e**) *herc3*; **f**) *herc6*; **g**) *ifng*; **h**) *viperin*; **i**) *sntb1*; **j**) *ctsl1*; **k**) *ctsf*

The qPCR results of pIC-responsive transcripts are presented based on their functions (e.g. receptors and transcription factors) in immune pathways (Figs. 5, 6, 7 and 8). These pIC-responsive transcripts were selected for qPCR validation from transcripts identified by both SAM and RP in both dietary groups (783 DEP; see Fig. 2), except for *cd209d* (RP-identified in the FO5 group), and *stat1* and *irf7* (SAM-identified in both diet groups). We chiefly aimed to include representative transcripts associated with different immune pathways (e.g. IFN, TLR and MAPK) and with different regulation (e.g. suppressed or induced) in qPCR assays to confirm our microarray results. Also, we subjected some microarray-identified transcripts (e.g. *sntb1*, *ctsf*, *optn*, *cflar* and *cytip*) to qPCR validation, as they were known to have immune- or macrophage-related functions in higher vertebrates but were not well-characterised in fish species (see Discussion for details and references). The microarray results were qPCR-validated for all of the pIC-responsive transcripts (for at least one of the dietary groups). However, no significant differences were found between the pIC responses of different dietary groups, except for *dusp22a* (*dual specificity phosphatase 22-a*) at 6 HPS (Fig. 6l). The expression results of pIC-influenced transcript are for both dietary groups unless otherwise noted.

We measured the expression of 10 transcripts (i.e. 8 microarray-identified transcripts as well as *tlr3* and *tlr7* that were absent in the microarray platform) playing roles as PRRs (pattern recognition receptors) or other receptors (Fig. 5). The expression of *lgp2* and *cxcr3* (*C-X-C chemokine receptor type 3*) was up-regulated in salmon MLCs in response to pIC at both 6 and 24 HPS, and increased significantly at 24 HPS within the pIC group in each diet (Fig. 5a and b). The up-regulation (i.e. more than 2-fold) of *cd209e* (*cd209 antigen-like protein e*) and *tlr7* (*toll-like receptor 7*) in pIC-stimulated salmon MLCs was only observed at 24 HPS (Fig. 5c and e). While no pIC induction was recorded for *tlr3* in salmon MLCs, this transcript showed a time-dependent up-regulation within the PBS groups of both diets at 24 HPS compared to 6 HPS (Fig. 5d). No early pIC response was seen for microarray-identified down-regulated transcripts with putative roles as receptors [i.e. *scarb1-a* (*scavenger receptor class B type I-like*), *scarb1-b*, *csf1r* (*macrophage colony stimulating factor 1, receptor 1*), *cmklr1* (*chemokine receptor-like 1*) and *cd209d*], and significant down-regulation was only found at 24 HPS for them (Fig. 5). There was a time-dependent up-regulation for *scarb1-a*, *scarb1-b* and *csf1r* in salmon MLCs within the PBS groups at 24 HPS compared to the earlier time point; in other words, pIC stimulation markedly suppressed the time-dependent response of these transcripts (Fig. 5f-h). Two different paralogues (i.e. 90% similarity at the nucleotide level) of salmon *scarb1* responded similarly to pIC (Fig. 5f and g). Nonetheless, the down-regulation of the *scarb1-a* (0.1-fold) in response to pIC was stronger than that of *scarb1-b* (0.4-fold), as seen in the microarray results [*scarb1-a* (probe ID: C089R130), 0.39-fold and *scarb1-b* (probe ID: C118R093), 0.47-fold].

Twelve pIC-responsive transcripts involved in signal transduction were subjected to qPCR validation (Fig. 6). The expression of *map3k8* (*mitogen-activated protein kinase kinase kinase 8*), *socs1* (*suppressor of cytokine signaling 1*), *socs3* and *dusp5* (*dual specificity phosphatase 5*) in salmon MLCs was significantly induced by pIC at both sampling points (Fig. 6a-d). Although *dusp5* response to pIC was unaffected by time, there was a decrease in *map3k8* expression as well as an increase in transcript levels of *socs1* and *socs3* within the pIC groups of both dietary treatments at 24 HPS compared to the earlier time point. The transcription of *traf5a* (*TNF receptor-­associated factor 5-­like a*), *jak3* (*tyrosine kinase JAK3*), *cytip* (*cytohesin-­interacting like*) and *ikka* (*inhibitor of nuclear factor kappa-B kinase subunit alpha*) increased more than 2.4-fold in pIC-stimulated MLCs at 24 HPS (Fig. 6e-h). Despite the time-dependent induction of *traf5a* in both PBS and pIC groups at 24 HPS, the up-regulation of this transcript was strengthened by pIC stimulation. The expression of *cd80*, *mapk13* (*mitogen-activated protein kinase 13*), *dusp6* and *dusp22a* was significantly reduced in pIC-stimulated salmon MLCs at 24 HPS (Fig. 6i-l). Additionally, *cd80*, *dusp6* and *dusp22a* were up-regulated in salmon MLCs within the PBS groups at 24 HPS compared to 6 HPS, but their expression was suppressed by pIC stimulation.

We measured the relative quantity of 5 pIC-responsive transcription factor encoding transcripts in salmon MLCs (Fig. 7). Salmon *crem* (*cAMP-­responsive element modulator-like*) was significantly induced by pIC at both time points (1.7- and 6.1-fold increase at 6 and 24 HPS, respectively), although its expression was significantly suppressed by sampling time within the PBS group at 24 HPS compared to 6 HPS (Fig. 7a). *stat1* (*signal transducer and activator of transcription 1*) and *irf7* (*interferon regulatory factor 7*) showed a time-dependent up-regulation within PBS and pIC groups (i.e. except for pIC group of FO5 in *irf7*) at 24 HPS, but the pIC-dependent up-regulation (at least 1.4-fold) of them was only significant in the FO7 group (Fig. 7e and b). The other studied transcription factors [i.e. *atf3* (*cyclic AMP-dependent transcription factor ATF-3*) and *batf3* (*basic leucine zipper transcription factor, ATF-like 3)*] were also positively regulated in pIC-stimulated MLCs at 24 HPS (Fig. 7c and d). The expression of *atf3* was significantly repressed within the PBS group of FO7 diet at 24 HPS compared to 6 HPS.

The results of 10 pIC-responsive transcripts, playing putative roles as immune effectors, were confirmed by qPCR analyses (Fig. 8). Also, *mx-b* was included in the qPCR study as a candidate diet-responsive transcript; however, it was not differentially expressed between dietary groups. The expression of the *rnf8* (*ring finger protein 8, E3 ubiquitin protein ligase*) and *cflar* (*CASP8 and FADD-like apoptosis regulator*) in salmon MLCs was significantly induced by pIC at 6 HPS (1.7- and 2.4-fold increase) and peaked at 24 HPS (2.4- and 3.4-fold increase; Fig. 8a and b). Similar results were seen for *mx-b*, *optn* (*optineurin*) and *herc3* (*E3 ubiquitin-protein ligase herc3*), but the significant differences between PBS and pIC treatments at the early time point were only observed for the FO7 group (Fig. 8c–e). Salmon *mx-b* was a time-responsive transcript in MLCs, as its expression increased within the PBS group over time. The expression of *herc6*, *ifng* (*interferon, gamma*), *viperin*, *sntb1* (*beta-1 syntrophin*) and *ctsl1* (*cathepsin-L1-like*) did not vary between PBS and pIC at 6 HPS (Fig. 8f-j), and they were up-regulated in response to pIC at 24 HPS (between 3.4- to 7.8-fold increase). Salmon *ctsf* (*cathepsin-f*) expression significantly increased within the PBS group at the latter time point, although it was significantly down-regulated by pIC at 24 HPS, compared to the time-matched PBS group or the pIC group at 6 HPS (Fig. 8k).

## Discussion

### Effects of experimental diets on cellular functions and fatty acid composition of MLCs

Neither phagocytosis nor RB of salmon MLCs varied between diets. Similarly, the phagocytosis of rainbow trout (*Oncorhynchus mykiss*) HKLs did not change with different levels of plant-based n-3 and n-6 in the diet [55]. In Seierstad et al. [21], RB and pro-inflammatory cytokine expression of HKLs remained unchanged in salmon fed different dietary levels of fish and vegetable oil. On the other hand, we observed some changes in lipid and fatty acid contents of salmon HKLs fed different diets. For example, linoleic acid (18:2n-6) and free fatty acids increased, but EPA (20:5n-3) decreased in the HKLs isolated from salmon fed FO5 diet compared to those fed FO7 diet (Table 2). In our study, the proportions of sterols and phospholipids significantly increased and decreased, respectively, in MLCs of the FO5 group; thus, the higher levels of dietary vegetable oil may influence the membrane structure of salmon MLCs. As in our study, in Seierstad et al. [21], the sum of n-6 fatty acids in salmon HKLs increased with a vegetable oil diet, although EPA + DHA levels decreased. However, the proportions of EPA + DHA in the experimental diets of the present study were lower than those of the previous study (i.e. at least 3.4% EPA + DHA in diet) [21]. The current study showed that feeding a diet containing 1% EPA + DHA for 16 weeks did not appear to influence cellular functions (i.e. phagocytosis and RB) and antiviral responses of MLCs. However, unbalanced levels of n-3 or n-6 can alter the immune or inflammatory responses of mammalian macrophages [6, 8, 9]. Hence, some diet-associated variations in gene expression of MLCs, discussed in the following paragraphs, may have arisen from the differences in fatty acid contents of the cells between dietary treatments.

### Impact of experimental diets on transcript expression of salmon MLCs

Hierarchical clustering analyses using the whole microarray dataset showed that most of the PBS control samples from the same dietary treatment (especially FO5) grouped closely together. The comparable constitutive global gene expression of the samples belonging to a dietary group may be explained by slight changes in the lipid and fatty acid content of HKLs. RP identified 54 and 14 DEP between the pIC and PBS groups of the two dietary treatments, respectively (Additional file 3: Table S2). However, 12 DEP between the PBS-matched groups overlapped with the DEP in the pIC-matched groups. Nine candidate diet-responsive transcripts identified by microarray analyses were subjected to qPCR validation, and the majority of them showed similar down- or up-regulation trends compared with the microarray results (Table 4). The expression of *psmb8* and *fabp4* significantly differed between the PBS- and pIC-matched groups of FO5 and FO7 (Fig. 4). The expression of *psmb8* was strongly suppressed in MLCs by lowering the level of fish oil in the diet. PSMB8 (alias LMP7) is an IFN (interferon)- and TNF-induced immunoproteasome subunit, involved in peptide processing of MHC-I pathway in antigen presenting cells (APCs) [56]. A previous study reported a significant up-regulation of *psmb8* and *MHC-I* in salmon HKLs after 3 days of stimulation with ISAV or pIC [57]. Lungfish (*Protopterus dolloi*) *psmb8* was also found to be an IFN- and pIC-induced gene [58]. In the present study, *MHC-I* expression significantly increased 1.4-fold in pIC-stimulated MLCs within the FO7 group at 24 HPS, and a non-significant up-regulation (i.e. 1.8-fold) was seen for *psmb8* in this group. However, these transcripts were not induced by pIC in the FO5 group. In addition to immunoregulatory functions, immunoproteasomes are suggested to eliminate oxidant-damaged proteins, resulting in cell protection against oxidative stress induced by immune responses [59, 60]. Further studies are needed to determine the correlation between dietary EPA/DHA and immune-derived oxidative stress with *psmb8* expression.

The qPCR assays in the present study did not validate the microarray results for *lgmn* (i.e. up-regulated in pIC group of FO5 at 24 HPS); however, qPCR showed that the expression of this transcript significantly increased in the pIC group of FO5 at 6 HPS compared to that of FO7. *lgmn* is associated with macrophage activity and differentiation in mammals [61, 62], and it has been shown to be more highly expressed in mature macrophages compared with less differentiated stages (early progenitors and monocytes) in goldfish [63]. If *lgmn* function is conserved in mammalian and teleost macrophages, then our *lgmn* expression results suggest that dietary fish oil (i.e. EPA/DHA) may influence salmon macrophage function.

As identified by microarray analyses and validated by qPCR, *fabp4* was significantly up-regulated in both the PBS and pIC groups of FO5 compared to those of FO7. FABP family proteins are lipid chaperones that regulate the specific lipid transfer to different compartments of the cells, thereby influencing cell signalling, lipid storage, membrane synthesis and lipid-mediated transcriptional control [64]. In mammals, different members of the FABP family are expressed in a tissue-specific manner, and *fabp4* is known to be transcribed in some immune-related cells such as macrophages and dendritic cells [64]. Mammalian FABP4, which plays a role in cell lipid transport of differentiated adipocytes and macrophages, was suggested to be a modulator of energy homeostasis [65]. Further, *fabp4*-deficient macrophages of mice developed impaired cholesterol trafficking, suppressed IKK (inhibitor of nuclear factor kappa-B kinase) signalling pathway and, consequently, decreased production of inflammatory cytokines [66]. Human macrophages were shown to up-regulate *fabp4* expression in response to PUFA oxidation via the mediation of Akt (protein kinase B)- and ERK (extracellular signal-regulated kinase)-dependent signalling pathways [67]. Importantly, FABP4 was established to increase the expression of inflammatory genes in human macrophages and to be involved in the development of atherosclerosis [68]. There is no information on *fabp4* functions in activity and lipid metabolism of fish macrophages. However, as in mammalian *fabp2* [64], the highest expression of Atlantic salmon *fabp2* was found in the intestine [69]. There was also a decrease in *fabp2* expression by intestinal inflammation caused by dietary soybean meal [69]. Collectively, these studies suggest a possible correlation between dietary-induced immune responses of Atlantic salmon and the expression of *fabp*s. In addition, it seems that *fabp4* may be a key gene in Atlantic salmon macrophage function, as in higher vertebrates. In the present study, the significant up-regulation of salmon *fabp4* in response to the higher level of dietary n-6 fatty acids may be influenced by PUFA-dependent responses of *fabp4*. We did not observe a significant difference in inflammatory biomarkers between the dietary groups in microarray analyses, although the larger number of microarray-identified pIC-responsive probes in the FO5 group compared to the FO7 group may be affected by the inflammation- or immune-related roles of *fabp4*. In the present study, the 4-fold up-regulation of *fabp4* occurred in response to a relatively small decrease in EPA + DHA content of the diet; therefore, a larger difference in dietary EPA + DHA or a longer feeding trial may increase the fatty acid-associated responses of *fabp4* and consequently its putative function in lipid transport of Atlantic salmon macrophages. Further studies are required to characterise *fabp4* in Atlantic salmon and to determine the fatty acid metabolism- and immune-related functions of this gene in Atlantic salmon macrophages.

In the present study, we used an ex vivo approach to determine dietary fatty acid-dependent transcriptomic responses in Atlantic salmon MLCs. The choice of an ex vivo model allowed us to evaluate the impact of dietary DHA + EPA on the cell type of interest (i.e. macrophages) and to assess the antiviral response and cellular function of each individual fish fed a given experimental diet. However, it is noteworthy that the antiviral immune response of fish assessed by in vivo studies may vary from these ex vivo experiments, due to different contributing factors such as paracrine signalling. Also, the cell isolation and culture procedures in the present study could influence the fatty acid composition of cell membranes, and potentially modulate the diet-associated responses of MLCs to immune stimuli. Still, since both diet groups of MLCs in this study were subjected to the same conditions for cell culture and immune stimulation, the observed changes in fatty acid composition and gene expression occurred in response to variation in the fatty acid composition of the diets. Our results show that 1% and 1.4% EPA + DHA diets for Atlantic salmon have different effects on the expression of some macrophage transcripts (i.e. *psmb8*, *fabp4* and *lgmn*) with putative roles in inflammation and/or macrophage function, thus suggesting them as important immune-related diet-associated biomarkers. Moreover, these differentially regulated transcripts, alongside the fatty acid composition results, suggest that a relatively small change in EPA + DHA intake may result in altered membrane lipids and gene expression in immune cells of salmon.

### Global transcript expression of pIC-stimulated salmon MLCs

Using SAM, we identified 3089 DEP responsive to pIC (i.e. 890 DEP overlapping with RP) within the FO7 group and 4745 DEP responsive to pIC (i.e. 1128 DEP overlapping with RP) within the FO5 group (Fig. 2). With respect to the DEP overlapping between SAM and RP, 612 and 705 DEP were up-regulated by pIC within the FO7 and FO5 groups, whereas 278 and 423 DEP were down-regulated by pIC within the FO7 and FO5 groups, respectively. The number of microarray-identified, pIC-responsive probes in the current study was higher than the previously published studies on ISAV-infected salmon MLCs [30, 31]. These differences may be influenced by the microarray platforms (i.e. 44K in the current study vs. 16K or 1.8K in previous studies) used in the different studies. Furthermore, the inter-study variation may arise from the differences in cell types (e.g. primary cell culture vs. cell line) and stimulating agents (e.g. viral mimic vs. viral pathogen) used in our study compared to the previously conducted investigations. As in the present study, RNA-seq analyses showed a massive gene expression response [i.e. 3149 differentially expressed genes (DEG)] in IFN-treated Atlantic salmon macrophage/dendritic-like TO cells [70]. Furthermore, the ratios of up-regulated to down-regulated transcripts by pIC in our study are similar to those of pIC-stimulated cod macrophages [71] and IFN-exposed salmon TO cells [70]. The higher number of DEP responsive to pIC in the FO5 group compared with the FO7 group could be influenced by biological variability in basal transcript expression and/or pIC response. We found a strong response to pIC for all of the microarray-studied samples, as samples belonging to the same stimulation group (i.e. PBS or pIC) clustered together.

### pIC-responsive transcripts with putative roles as PRRs or other receptors

**Fig. 9:**
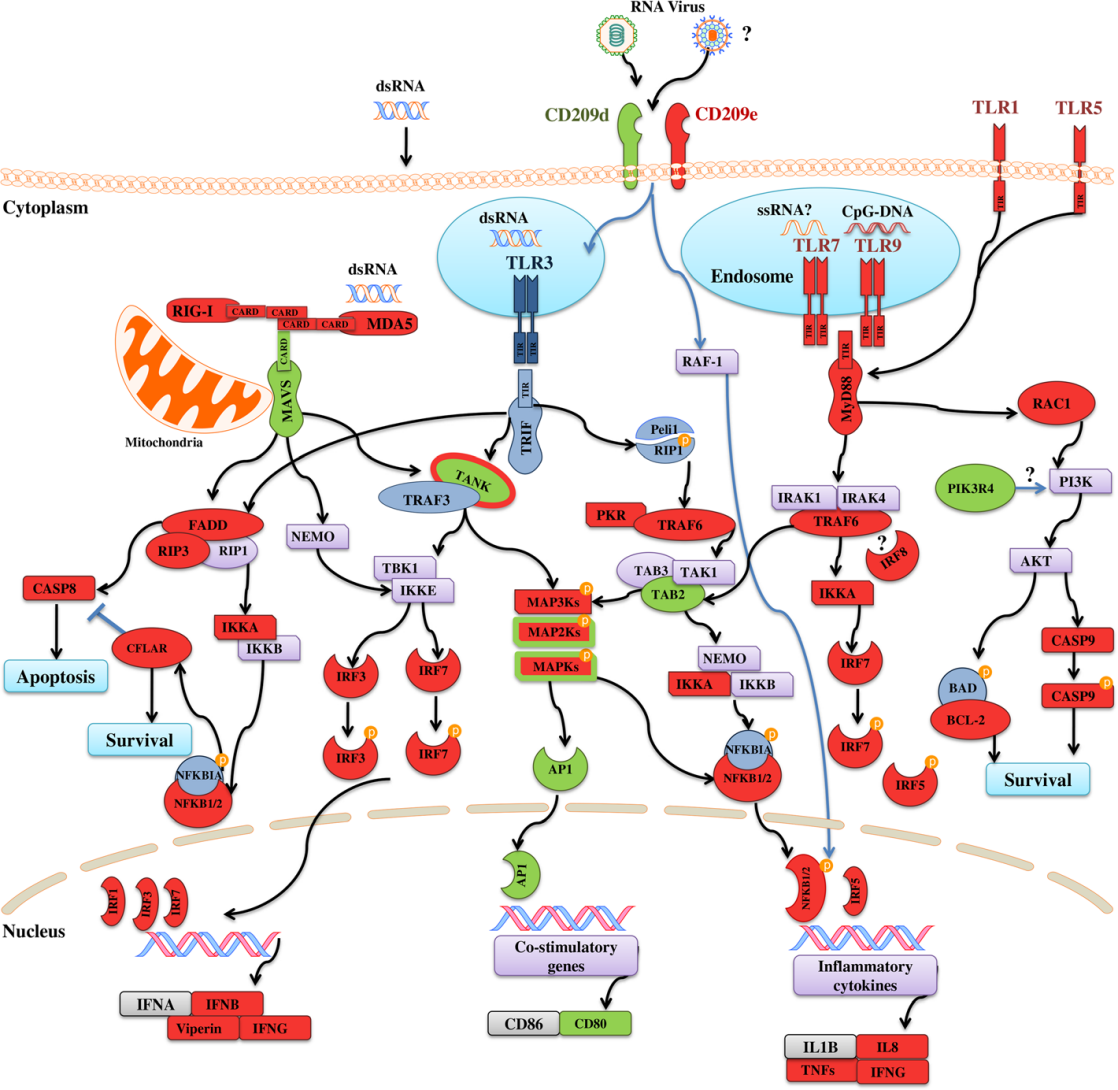
The activated PRRs and signalling pathways by pIC in Atlantic salmon MLCs. This figure was adapted from known mammalian pathways [79–81, 83, 97, 99, 129, 131]. A question mark below or above the gene/protein name indicates putative regulating mechanism in mammals. The up- and down-regulated transcripts by pIC in the present study are drawn in red and green, respectively. The genes/proteins drawn in both red and green represent both up- and down-regulation of different probes associated with them. The microarray result of *irf7* was only qPCR-validated for the FO7 group. MDA5 (melanoma differentiation-associated protein 5), RIG-I (retinoic acid-inducible gene), LGP2 (RNA helicase LGP2), MAVS (mitochondrial antiviral-signaling protein), FADD (FAS-associated death domain), RIP1/3 (receptor-interacting protein 1/3), IKK (inhibitor of nuclear factor kappa-B kinase), NFKB1/2 (nuclear factor kappa-B 1/2), NFKBIA (NF-kappa-B inhibitor alpha), CASP (caspase), CFLAR (casp8 and fadd-like apoptosis regulator), TNF (tumour necrosis factor), IL (interleukin), IFN (interferon), NEMO (NFKB1 essential modulator or IKKG), TLR (Toll-like receptor), TRIF (TIR domain-containing adaptor protein inducing IFNB), TRAF (TNF receptor-associated factor), TANK (TRAF family member-associated NFKB activator), TBK (tank-binding kinase), IRF (IFN regulatory factor), MAP3K (mitogen-activated protein kinase kinase kinase), MAP2K (dual specificity mitogen-activated protein kinase kinase), MAPKs (mitogen-activated protein kinase), AP1 (transcription factor AP1), Peli1 (pellino E3 ubiquitin protein ligase 1), PKR (IFN-induced, double-stranded RNA-activated protein kinase), TAK1 [transforming growth factor beta (TGFB)-activated kinase 1 or MAP3K7], TAB (TAK1-binding protein), RAF-1 (serine/threonine kinase Raf-1), MyD88 (myeloid differentiation primary response gene 88), IRAK (IL-1 receptor-associated kinase), RAC1 (ras-related C3 botulinum toxin substrate 1), PI3K (phosphoinositide 3-kinase), PIK3R4 (phosphoinositide 3-kinase regulatory subunit 4), AKT/PKB (protein kinase B), BAD (Bcl-2-associated death promoter). Orange circles show phosphorylation

As identified by microarray analyses and validated by qPCR, pIC stimulation changed the expression of several transcripts encoding PRRs and other receptors in salmon MLCs. The expression of *tlr9* (identified by microarray) and *tlr7* (studied by qPCR), known as the endosomal PRRs activating the MyD88-dependent pathway, was up-regulated by pIC in salmon MLCs. Mammalian TLR7 and TLR9 are responsible for recognising ssRNA viruses and CpG-rich bacterial DNA/dsDNA viruses, respectively (Fig. 9) [72, 73], yet their functions are poorly understood in fish species [74, 75]. Atlantic salmon TLR9 was shown to bind with synthetic oligonucleotides but in a CpG-independent manner, indicating the evolutionarily conserved feature of TLR9 binding to DNA [76]. TLR3 is the main PRR detecting dsRNA in mammals and fishes [72, 75], even though the expression of its encoding transcript was not affected by pIC in salmon MLCs in the current study. This expression pattern was similar to *tlr3* in the spleen of pIC-injected Atlantic cod [77] and was in disagreement with Salmonid alphavirus (SAV)-infected TO cells [70]. Nevertheless, the GO terms associated with TRIF (TIR-domain-containing adaptor protein inducing IFNB)-dependent TLR and TLR3 signalling pathways were over-represented in the pIC-responsive transcript list of the present study, revealing the activation of the TRIF-dependent pathway by pIC downstream of TLR3 (see Fig. 9 for pIC-activated signalling pathways).

We found the up-regulation of RLR (RIG-I-like receptors) family members (i.e. *rig-i* alias *ddx58*, *lgp2* alias *dhx58*, and *mda5* alias *ifih1*) in pIC-treated salmon MLCs (Additional file 4: Table S3), similar to that reported in TO cells 48 h post-exposure to SAV [70] and RTG-2 cells (i.e. rainbow trout fibroblast-like cell line) 24 h after pIC stimulation [78]. MDA5 and RIG-I are involved in mitochondrial-dependent recognition of dsRNA and ssRNA viruses in the cytoplasm (Fig. 9), whereas LGP2 plays roles as a positive or negative regulator of other RLRs [79–81]. Fish MDA5 and RIG-I have been suggested to exhibit evolutionarily conserved functions, but the molecular function of LGP2 in fishes is not fully understood [82]. The up-regulation of *lgp2* by pIC at 6 HPS observed herein shows the importance of this transcript in the early antiviral responses of salmon MLCs.

We identified some pIC-responsive transcripts that can facilitate the entrance of pathogens into cells. In this study, *cd209e* expression increased over time within both the pIC and PBS groups, and it was also up-regulated (more than 3-fold) in response to pIC at 24 HPS. Conversely, *cd209d* was down-regulated by pIC at 24 HPS. CD209 acts as a PRR and facilitates the entry of pathogens into the endosomes, resulting in activation of MHC-I-dependent antigen presentation; additionally, it modulates the TLR-dependent signalling pathway and promotes the DNA affinity of NFKB [83, 84]. Zebrafish *cd209* was shown to be associated with several APCs and an important gene for adaptive immunity [85]. The distinct regulation of salmon *cd209e* and *cd209d* by pIC stimulation seen in the present study suggests that these transcripts have distinct functions in immune responses of salmon MLCs.

qPCR analyses showed a time-dependent up-regulation for *csf1r* and both paralogues of *scarb1* within the PBS groups, and significant suppression by pIC at 24 HPS. Mammalian SCARB1 is a high-density lipoprotein (HDL) receptor that changes the cholesterol content of cell plasma membranes via mediating in lipid transfer, but it can also be employed as a co-receptor for viral internalisation into the host cells [86, 87]. Similar to our findings, zebrafish CD36 (a family member of SCARB) was down-regulated in response to bacterial infection [88]. CSF1R is an important biomarker for teleost fish macrophage maturation [89]; therefore, the present results suggest a suppressed macrophage maturation in the pIC group over time. Also, the down-regulation of salmon *csf1r* in pIC-stimulated MLCs may be attributed to SOCS1 (i.e. a pIC-induced transcript in our study; Fig. 6b), as described for other fish species [89]. A soluble isoform of teleost CSF1R was found to be a regulator of inflammatory cytokines [90]. The different isoforms of CSF1R in salmon macrophages are yet to be structurally and functionally characterised.

The present investigation identified several pIC-responsive chemokine receptors in salmon MLCs (Additional file 4: Table S3). There was an up-regulation of salmon *cxcr3* in pIC-stimulated MLCs in the present study, and teleost *cxcr3* (e.g. common carp, *Cyprinus carpio*) was previously reported to be a MCSF (macrophage colony-stimulating factor)- and IFNG-induced transcript involved in macrophage trafficking and macrophage-mediated responses [91, 92]. Contrary to the results for *cxcr3* in the present study, pIC strongly repressed *cmklr1* in salmon MLCs at 24 HPS (Fig. 5i). Mammalian CMKLR1 is a well-established molecule mediating macrophage adhesion and migration as well as inflammatory responses [93], but its role in fish macrophages remains undescribed. In our study, the transcript expression results (i.e. positive or negative regulation), along with over-representation of GO terms associated with chemokine receptor activity and chemokine-mediated signalling pathway (see Table 3), reveal the importance of different chemokine receptors in antiviral immune responses of salmon MLCs.

### pIC-responsive transcripts involved in signal transduction and transcriptional regulation

**Fig. 10:**
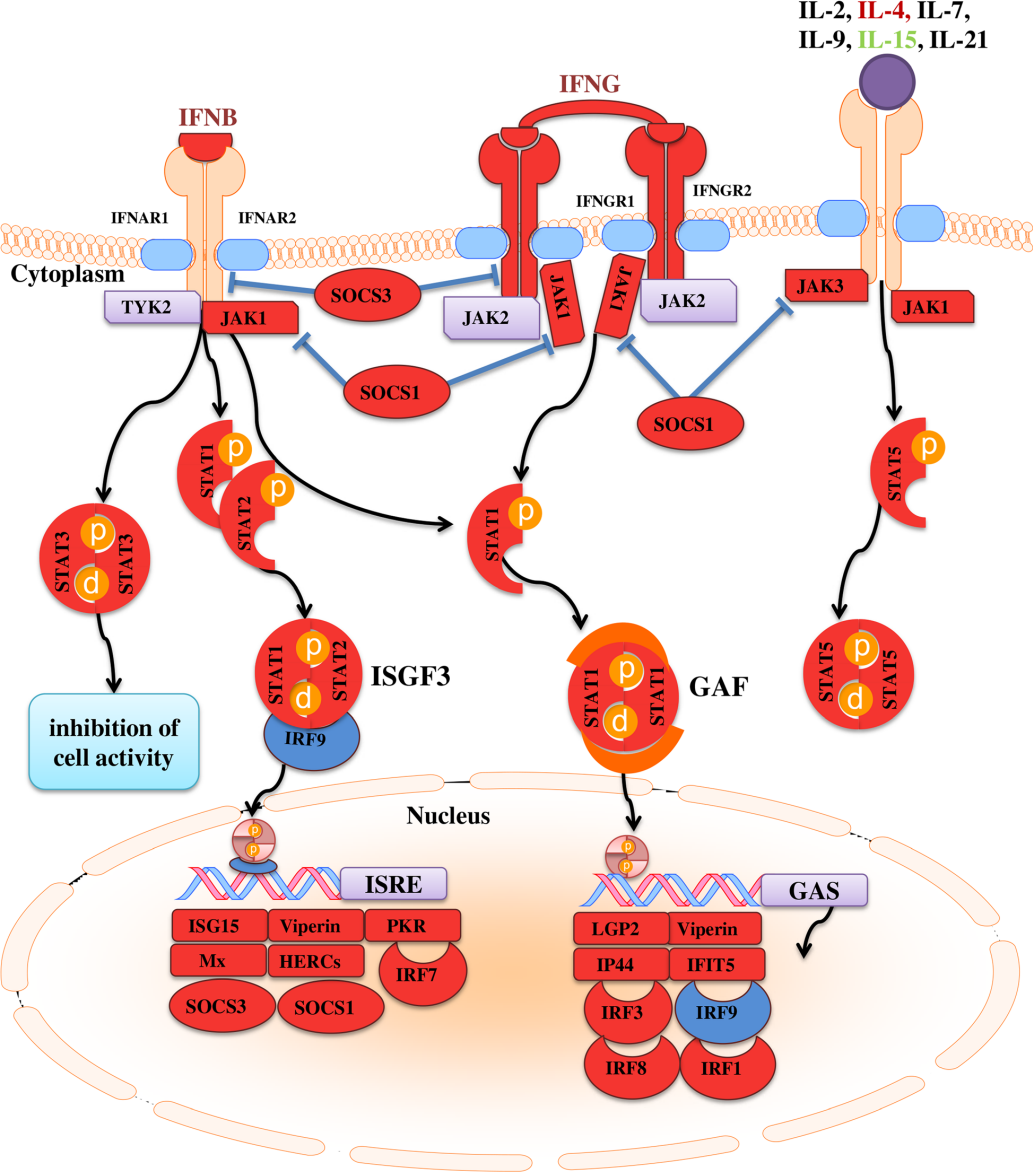
The cytokine-mediated pathways by pIC in Atlantic salmon MLCs. This figure was adapted from known mammalian pathways [79, 97, 107–109]. The up- and down-regulated transcripts by pIC in the present study are drawn in red and green, respectively. The microarray results of *stat1* and *irf7* were only qPCR-validated for the FO7 group. IFNB/G (interferon beta/gamma), IFNAR (IFN alpha receptor), IL (interleukin), IFNGR (IFN-gamma receptor), TYK2 (tyrosine kinase 2), JAK (Janus kinase), SOCS (suppressor of cytokine signaling), STAT (signal transducer and activator of transcription), IRF (IFN regulatory factor), ISGF3 (IFN-stimulated gene factor 3), ISRE (IFN-sensitive response element), GAF (IFNG-activated factor), GAS (IFNG-activated sequence), ISG15 (IFN-stimulated gene 15), LGP2 (RNA helicase LGP2), IP44 (IFN-induced protein 44-like), HERC (E3 ubiquitin-protein ligase herc), IFIT5 (interferon-induced protein with tetratricopeptide repeats 5)﻿. Orange circles show phosphorylation

The current study identified a large number of pIC-responsive transcripts involved in signal transduction and transcription control. The qPCR analyses showed both early and late up-regulation responses to pIC for several transcriptional regulators (i.e. *map3k8*, *socs1*, *socs3*, *dusp5*, *crem* and *irf7*), whereas other studied signal transductors and transcription factors (i.e. *traf5a*, *jak3*, *cytip*, *ikka*, *atf3*, *batf3* and *stat1*) were only up-regulated by pIC at 24 HPS (Figs. 6 and 7). The qPCR assays also revealed the suppressed expression of *cd80*, *mapk13*, *dusp6* and *dusp22a* by pIC stimulation at 24 HPS. As illustrated in Fig. 9, the pIC stimulation of salmon MLCs activated the MAVS (mitochondrial antiviral-signalling protein)-, TRIF- and MyD88-dependent signalling pathways downstream of RLRs and TLRs. As shown in Fig. 9, opposite to the induction of *rig-i* and *mda5*, the expression of *mavs*, which plays a role as their adaptor, was down-regulated by pIC in salmon MLCs. The RLR pathway and MAVS activity seem to be conserved between fish and mammalian species [94]. Human *mavs* was down-regulated in pIC-stimulated glial cells, and its knockdown was associated with suppression of inflammatory cytokines [95]. Accordingly, the inhibition of *mavs* in the present study may be related to the immunoregulatory functions of this gene. In addition to this pathway, our microarray results (Additional file 4: Table S3) revealed an up-regulation of transcripts encoding signalling adaptors (e.g. TRAF6) and kinases [e.g. PKR (IFN-induced, double-stranded RNA-activated protein kinase)] that are known to trigger a series of events activating transcription factors. IKKs phosphorylate NFKB inhibitor, resulting in translocation of NFKBs into the nucleus and production of cytokines and inflammatory proteins (reviewed by [96]). The qPCR results for *ikka* expression (i.e. 3-fold up-regulation at 24 HPS), along with the identification of *nfkb1/2* as pIC-responsive transcripts by microarray analyses, indicate the importance of NFKB-related transcription responses in the antiviral mechanisms of salmon MLCs. In agreement with a previous study on pIC-induced cod macrophages [71], members of the IRF family (e.g. *irf1*, *irf3* and *irf7*) were slightly up-regulated (1.4- to 1.7-fold) by pIC in salmon MLCs. IRF7 and IRF3, known as the main family members involved in virus-associated responses, boost the transcription of *ifn*s and IFN-sensitive response element (ISRE)-containing genes (see Figs. 9 and 10), following phosphorylation by IKKA or IKKE [97, 98]. Additionally, other microarray-identified IRFs (e.g. *irf8*) in this study were previously shown to promote IFN induction of mammals by interacting with adaptors in the MyD88-dependent pathway (see Fig. 9) (reviewed by [98]).

MAPK-dependent induction by the TLR pathway can play crucial roles in the innate immune and inflammatory responses [99]. The current microarray analyses identified multiple pIC-regulated transcripts at different levels of the MAPK cascade (see Fig. 9), suggesting the activation of this pathway in innate antiviral immune responses of salmon MLCs. As validated by qPCR, *map3k8* was up-regulated by pIC at both the early and the late time points; the mammalian orthologue of this transcript was found to regulate antiviral responses via IRF3 phosphorylation [100]. MAPK13 (alias p38 delta), a kinase involved in inflammatory responses, stimulates important transcription factors such as AP-1 in mammalian macrophages [101]. Therefore, the co-down-regulation of *mapk13* and *ap-1* by pIC (Fig. 9) seen herein suggests that *mapk13* function may be conserved in fish and mammalian macrophages. The activation of MAPKs is also managed by the DUSPs via negative feedback loops [100], and our study revealed the negative (e.g. *dusp22a* and *dusp6*) or positive (e.g. *dusp5*) regulation of different members of DUSP family in pIC-stimulated MLCs. In agreement with the current findings, a previous microarray analysis identified *dusp5* as a CpG- and LPS-induced transcript in mononuclear phagocytes of Atlantic salmon [102]. Mammalian *dusp5* is an LPS- and MCSF-induced gene that can restrict macrophage differentiation [100]. Similar to Atlantic salmon MLCs, pIC repressed the expression of *dusp6* in mammalian [103] and rainbow trout macrophages [104], suggesting that *dusp6*’s role in inactivating MAPKs of macrophages may be conserved between fish and mammals. DUSP22 was shown to be a negative regulator for STAT3 in cancer cells [105], but its role in antiviral responses is undetermined. While it appears that MAPKs and their regulating factors are crucial parts of antiviral responses of salmon MLCs, the precise functions of genes in this pathway are yet to be determined for fish species.

The current microarray-identified and qPCR-validated transcripts (e.g. up-regulation of *ifng*, *jak3* and *traf5a*), as well as our GO enrichment results (e.g. over-representation of cytokine/chemokine-mediated signalling pathway), reveal the IFN-triggered responses of salmon MLCs by pIC stimulation. As depicted in Fig. 10, IFNG and IFNB elicit the antiviral immune responses by up-regulating IFN-induced genes [97, 106]. In addition to *ifng* and *ifnb,* in the current study, pIC stimulation of salmon MLCs led to the co-up-regulation of *ifngr1*/*2* as well as kinases (i.e. *jak1*) and transcription factors (i.e. *stat1*, *stat2* and *stat3*) in the IFN pathway (Fig. 10). Similar trends were reported for salmon *jak1*, *stat1* and *stat2* in IFN- and SAV-infected TO cells [70]; correspondingly, the pIC-activated IFN pathway in salmon MLCs is assumed to be conserved with higher vertebrates. Mammalian JAK3 and TRAF5 are well-established as kinases associated with receptors of several cytokines (e.g. IL-2) [107] and as a signalling adaptor in cytokine-activated pathways (e.g. IL-17) [108], respectively. The pIC-associated induction of *traf5a* and *jak3* alongside the other microarray-identified pIC-responsive cytokines in this study (e.g. *il4*, *il12* and *stat5*; see Additional file 4: Table S3) suggest the importance of cytokine-activated pathways in the antiviral responses of salmon MLCs. Additionally, our qPCR analyses showed an up-regulation in immune-regulating factors (i.e. *socs1*/*3*, *crem*, *atf3* and *batf3*; Figs. 6 and 7). Mammalian SOCS1 and SOCS3 bind to chemokine receptors and JAKs, thereby inactivating JAK/STAT signalling [109]. These genes were also shown to be LPS- and CpG-DNA-inducible in mammalian macrophages as well as a contributing factor in PAMP-induced hypersensitivity [109]. The induction of *socs1* and *socs3* by pIC in the current study, together with similar results previously reported for SAV-exposed TO cells [70], suggest that *socs1/3* of Atlantic salmon macrophages may display a conserved function with their orthologues in higher vertebrates (see Fig. 10). Multiple alternatively spliced CREMs bind to promoters of cytokine genes, provoking the gene repression or activation through methylation-dependent mechanisms (reviewed by [110]). The regulatory role of BATF3 is chiefly linked to the development of APCs [111]. ATF3 was found to control IFN signalling and to repress PAMP-stimulated cytokine responses in mammalian macrophages [112, 113]. Consistent with the present study, Feng and Rise [114] characterised *atf3* as an evolutionarily-conserved and pIC-inducible transcript in Atlantic cod. Although we showed the involvement of cAMP-dependent factors such as *atf3* in the antiviral responses of salmon MLCs, the precise functions of these factors in teleost macrophages are yet to be investigated.

We found a down-regulation in *cd80* of salmon MLCs in response to pIC, but it was up-regulated in trout leukocytes following LPS stimulation [115]. CD80 is a co-factor on the surface of APCs that regulates T-cell proliferation through engagement with CD28 [116]. Although CD80 is not a well-characterised protein in fish species, it has been shown to be functionally and structurally conserved in rainbow trout [115]. Our results suggest that the transcriptional regulation of salmon *cd80* may be similar to that of higher vertebrates since there was a co-down-regulation by pIC for *cd80* and *ap-1* in this study (Fig. 9; Additional file 4: Table S3). In contrast to *cd80*, the expression of *cytip* (alias *pscdbp*) increased by pIC in salmon MLCs (Fig. 6g). Mammalian CYTIP regulates T cell-APC adhesion in lymphocytes [117]. It appears that pIC stimulation changes the expression of the genes involved in the antigen presenting function of salmon macrophages.

### pIC-responsive transcripts with putative functions as immune effectors

Figure 8 represents a subset of immune effectors activated through PRR- or IFN-mediated pathways in salmon MLCs. Mammalian RNF8 is responsible for ubiquitination of H2A in response to DNA damage [118]. Interestingly, some viruses target RNF8 via phosphorylation-based degradation to enhance viral replication/transcription [118], thus indicating the importance of RNF8 in the virus-host battle. Our transcript expression results, along with the over-representation of histone H2A ubiquitination process in the pIC gene list, may reflect the activation of the DNA repair pathway in salmon during the antiviral response. Additionally, we found a significant induction in pIC-exposed salmon MLCs for *herc4* (2-fold increase; only microarray-identified), *herc3* and *herc6*, members of a protein family containing HECT and RCC1 domains. Likewise, *herc4* expression increased in pIC-stimulated cod macrophages [71] and ISAV-exposed salmon MLCs [30]. Different HERCs (i.e. HERC5 in human or HERC6 in mice) in higher vertebrates were reported as IFN-responsive and E3 ligase proteins that play roles in the ISGylation process via interaction with ISG15 [119, 120], thereby inhibiting viral replication. Despite the species-dependent E3 ligase activity of different HERCs, it remains unknown whether the members of this family in fish mediate ISGylation. As in the present study, there was an up-regulation of *mx* and *viperin* in rainbow trout monocyte/macrophage cells exposed to Chum salmon reovirus (CSV) [121], ISAV-stimulated salmon MLCs [30] and IFN-induced salmon TO cells [122]. Similar to its mammalian orthologue, the induction of fish Viperin occurs through the dsRNA-stimulated RLR pathway [123]. Mammalian Viperin restricts viral replication via an unknown molecular mechanism [124]. Furthermore, mammalian Mx exhibits antiviral functions against several RNA viruses (e.g. interference with viral genome replication) [125], and a previously published study confirmed the inhibition of infectious pancreatic necrosis virus (IPNV) replication with salmon Mx [126]. Collectively, the present study suggests that pIC activates the PRR- and IFN-dependent antiviral agents in salmon MLCs.

This investigation showed that *sntb1* is induced (more than 5-fold increase) in pIC-treated salmon MLCs at 24 HPS. SNTB1 is documented to modulate mammalian macrophage lipid efflux [127], but its function in antiviral responses is not well-understood. The qPCR assays showed a slight up-regulation for *optn* in pIC-exposed salmon MLCs (1.5-fold); this was, however, a lower fold-change than that seen in the microarray results. Mammalian OPTN is a virus- and pIC-induced protein that can inhibit the virus-induced IFNB production [128]. We revealed herein the activation of several apoptosis-related factors in pIC-stimulated salmon MLCs. Nonetheless, this induction was seen for both pro-apoptotic [e.g. *casp8* (caspase 8) and *casp9*] and anti-apoptotic (e.g. *bcl2*) agents (Fig. 9; Additional file 4: Table S3). The expression of *ctsl1* increased more than 6-fold in pIC-triggered salmon MLCs at 24 HPS, whereas there was a down-regulation (0.4-fold decrease) of *ctsf* in salmon MLCs by pIC. Similarly, *ctsa* expression was lowered by pIC in cod macrophages [71]. CTSs can facilitate cell death by means of degradation of the anti-apoptotic proteins or activation of granule-mediated apoptosis [reviewed by 129]. Moreover, CTSF influences the MHC-II pathway in macrophages via processing of Ii (invariant chain) [130]. The suppression of *ctsf* by pIC in the present study may be due to the involvement of this gene in a different molecular pathway (e.g. MHC-II). The expression of *cflar* was positively regulated by pIC at both early (more than 2-fold) and late (more than 3-fold) time points. CFLAR (alias cFLIP) controls cell apoptosis in mammals by inhibiting the CASP8-mediated pathway [131], but its function is not well-understood in fish species. More studies are needed to determine the PAMP-mediated regulation of apoptosis pathways in salmon MLCs.

## Conclusions

We used various cellular and molecular approaches to determine the effects of different dietary proportions of fish and vegetable oils on the antiviral immune responses of salmon MLCs. Although the fatty acid compositions of the diets did not influence the cellular functions of salmon MLCs, they changed lipid class and n-3 and n-6 proportions of HKLs. The variation in the fatty acid composition of the cells observed herein may have caused diet-associated regulation of gene expression. In addition, the lower level of EPA + DHA (i.e. 1% vs. 1.4%) in the diet influenced the expression of some genes in salmon MLCs. The up-regulation of *fabp4* and *lgmn*, with putative inflammatory- or macrophage-related functions, in the higher vegetable oil diet group in this study suggests immunomodulatory effects of dietary n-6 fatty acid level on salmon macrophages. This study suggests *fabp4* and *psmb8* are important diet-responsive immune-related biomarkers for future studies. However, the results of the current ex vivo-based study do not necessarily reflect the dietary fatty acid-associated responses of different tissues in Atlantic salmon at various ages. Thus, further in vivo and ex vivo-based investigations using a wider range of levels of dietary EPA + DHA, as well as various tissues and life stages, are suggested to broaden the current knowledge of immunomodulatory effects of dietary n-3 and n-6 fatty acids in salmon. The pIC-stimulated transcripts identified by microarray and validated by qPCR provide a better understating of the molecular pathways activated by the antiviral response in salmon MLCs. These results showed that different TLR- and RLR-dependent signalling pathways (e.g. IRFs, NFKB, and STATs) are stimulated by pIC. Further, the present results indicate the importance of MAPKs and their associated regulators in signal transduction of PRR- and cytokine-mediated pathways in salmon. We also identified several IFN-induced immune effectors (e.g. *viperin* and *herc6*), which may play roles in the inhibition of viral replication. Since the current study utilised a viral mimic rather than a live pathogen, further studies are required to evaluate the effects of dietary EPA + DHA on salmon MLC responses to viral infections. Moreover, the pIC-responsive genes identified in the present investigation should be functionally characterised to have a more comprehensive picture of their mechanistic roles in antiviral responses of salmon.

## Additional files

Additional file 1: Figure S1. Overview of experimental design. (PDF 580 kb)

Additional file 2: Table S1. Primers used in qPCR studies. (PDF 144 kb)

Additional file 3: Table S2. The diet-responsive probes identified by RP (PFP < 0.05) between PBS- and pIC-matched groups of dietary treatments. (XLSX 21 kb)

Additional file 4: Table S3. Complete list of significantly pIC- responsive probes identified by SAM (FDR < 0.05) and RP (PFP < 0.05) within each dietary group. (XLSX 788 kb)

Additional file 5: Figure S2. Hierarchical clustering analyses of samples based on of pIC-responsive transcripts overlapping between SAM- and RP-identified lists. (PDF 503 kb)

Additional file 6: Figure S3. GO term annotation of pIC-stimulated transcripts in different dietary groups. (PDF 457 kb)

Additional file 7: Table S4. The enriched GO terms of pIC-responsive transcripts (overlap between SAM and RP analyses) within each dietary group (Fisher’s exact test, FDR < 0.05). (PDF 250 kb)
