# Supplementary material for: Transcriptome profiling of antiviral immune and dietary fatty acid dependent responses of Atlantic salmon macrophage-like cells
Source: BMC Genomics. 2017 Sep 8;18:706. doi: 10.1186/s12864-017-4099-2 (PMC5591513; doi:10.1186/s12864-017-4099-2)

Supplemental Figure S1. Overview of experimental design.

Following 16 weeks of feeding trial, macrophage-like cells (MLCs) were isolated from 8 Atlantic salmon in each dietary group, and the isolated cells from each individual were exposed to PBS or 10 μg ml^{-1} pIC for 6 h or 24 h. The diet- and pIC-responsive transcripts were determined by the means of microarray analyses on 24 h time point samples. Subsets of microarray-identified transcripts were subjected to qPCR assays, using all samples at both time points. Phagocytosis and respiratory burst assays were conducted on MLCs, whereas the lipid and fatty acid analyses were performed on head kidney leucocytes (HKLs).

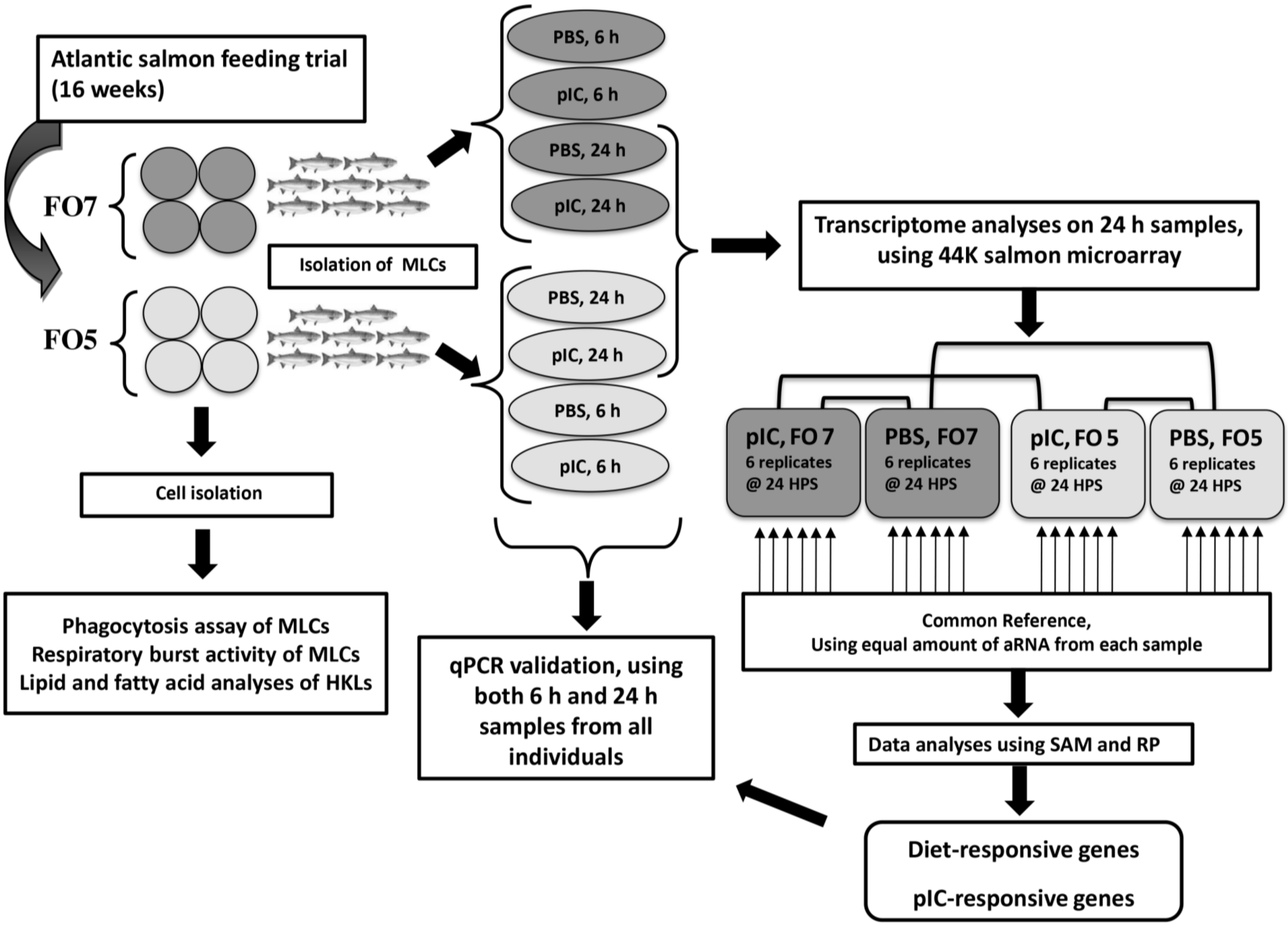

Supplement: Supplementary file 1 — Overview of experimental design. (PDF 580 kb) [file 12864_2017_4099_MOESM1_ESM.pdf]
