# Supplementary material for: Transcriptome profiling of antiviral immune and dietary fatty acid dependent responses of Atlantic salmon macrophage-like cells
Source: BMC Genomics. 2017 Sep 8;18:706. doi: 10.1186/s12864-017-4099-2 (PMC5591513; doi:10.1186/s12864-017-4099-2)
Supplement: Supplementary file 2 — Primers used in qPCR studies. (PDF 144 kb) [file 12864_2017_4099_MOESM2_ESM.pdf]

Supplemental Table S1. Primers used in qPCR studies

Gene name	GenBank accession number		Primer sequence 5' to 3'	R^2	Amplification efficiency (%)	Amplicon size (bp)
**Diet-responsive transcripts**						
Fc receptor-like protein 2 (*fcr2*)	DY734226	Forward	TCTTCATGCACCGTGTCACT	0.993	91.2	148
		Reverse	CTCCCTCAGTCACAGGAAGG			
fatty acid-binding protein, adipocyte (*fabp4*)	NM_001141203	Forward	GACTTGGGACGGCAAGACTA	0.992	97.9	128
		Reverse	CAGCAGACTGGAATCACACC			
FAD-linked sulfhydryl oxidase ALR-like (*fadox*)	GE791133	Forward	TTAGGGCGTAACACCTGGTC	0.998	102.3	144
		Reverse	CAGGTCCTCAGCACACTCCT			
legumain-like (*lgmn*)	EG917238	Forward	AGTCCGCTCCTCAAACTCAA	0.997	100.8	184
		Reverse	TGCTGTACCTGGTTTTGCTG			
lathosterol oxidase (*sc5d*)	NM_001140116	Forward	TGGCGACAACACTCACAGAT	0.992	103.9	172
		Reverse	TGAGCAAGGCACTCAACTCA			
MHC-I	AF504022	Forward	CATGAAGATGTGGAGCATGG	0.982	101.0	131
		Reverse	AGACCCGTGACTTGAACCAC			
proteasome subunit beta type-8 (*psmb8*)	BT058447	Forward	TACGCCTATGGTGTGATGGA	0.993	100.2	189
		Reverse	GTACAGGTCGCCAACATCCT			
tropomodulin-4-like (*tmod4*)	DW569648	Forward	GCTACCACTTCACCCAGCAG	0.994	98.1	153
		Reverse	CAAGGGGCTGAGTTGATTGT			
phospholipase d4 (*pld4*)	GE792176	Forward	GCAGTCCAACAGGCTTACTGT	0.998	102.2	159
		Reverse	ATCTCGCTGACTTGGGTCAT			
**pIC-responsive transcripts**						
**Receptors**						
RNA helicase lgp2 (*lgp2*)[a]	BT045378	Forward	TCCAAGACCCGTAAAAGCAC	0.999	89.1	189
		Reverse	GGTGGAGATCAGGAGGTTGA			
C-X-C chemokine receptor type 3 (*cxcr3*)	NM_001140493	Forward	GGTGTTGGTGCTGGTCTTTT	0.998	90.9	150
		Reverse	GCGAACGTAACCACCAGACT			
CD209 antigen-like protein e (*cd209e*)	NM_001141449	Forward	ATGTACTGGATCGGCCTGAG	0.998	93.6	109
		Reverse	AACCACAATCCCCTTGTCTG			
toll-like receptor 3 (*tlr3*) [a]	BK008646	Forward	AATATGGCGCTGGTGAAGAG	0.997	92.5	135
		Reverse	CGCAAAGGTGAACACTGAGA			
toll-like receptor 7 (*tlr7*) [a]	HF970585	Forward	CACCAACACAGAGCTGGAGA	0.994	98.3	184
		Reverse	GCCTTGGAAAACTTGCTGAG			
scavenger receptor class B type I-like (*scarb1-a*)	NM_001204894	Forward	AGGGGATAATGGAGGGGATT	0.995	92.1	153
		Reverse	ACACTGGGGAGTTGGATCTG			
scavenger receptor class B type I (*scarb1-b*)	NM_001123612	Forward	TTCCCAACCCCACTTCTACA	0.996	90.3	128
		Reverse	ACACATTCAGAGGCACACCA			
macrophage colony stimulating factor 1, receptor 1 (*csf1r*)	CB515019	Forward	GGTTGAGGAGTTGGAGCTGT	0.998	91.2	174
		Reverse	TTGATGATGTCGGAGCTGTC			
chemokine receptor-like 1 (*cmklr1*)	BT044928	Forward	ATGTTCAGCAGCGTCTTCCT	0.999	80.2 [b]	150
		Reverse	TACAGTCAGGGCAGCAGAGA			
CD209 antigen-like protein d (cd209d)	BT048497	Forward	AGAGAGGAGCAGACCTGGTG	0.993	88.7	187
		Reverse	GCACCATTATCAGGCTGGTT			
**Signal transduction**						
mitogen-activated protein kinase kinase kinase 8 (*map3k8*)	NM_001173785	Forward	GGTGAACGTGTGACTGATGC	0.996	99.7	144
		Reverse	GGCAGCTACAGAAACCACCT			
suppressor of cytokine signaling 1 (*socs1*)	EG924375	Forward	CTGTAGGATGGTCGCTCACA	0.999	94.4	133
		Reverse	ACACTGTTTGGATGGGTGCT			
suppressor of cytokine signaling 3 (*socs3*)	GE794538	Forward	ATGGTAGCCCACAGCAAGTT	0.997	89.2	179
		Reverse	GCCTCCTTGCCATTGATAGA			
dual specificity phosphatase 5 (*dusp5*)	BT049175	Forward	CCGTCGCCTCAAGAAGATTA	0.995	91.3	131
		Reverse	GGACAACAACCGAGTTCAGG			
TNF receptor-associated factor 5-like a (*traf5a*)	DY720479	Forward	AGAGGGGCCGTAAAGAACTG	0.997	98.2 [b]	140
		Reverse	GCATGGACGTGCTGACACTA			
tyrosine kinase JAK3 (*jak3*)	DY728848	Forward	TTCCCTCTCATCTTGGATGC	0.998	98.1	121
		Reverse	ACTCTGCCCTCCCAGAATTT			
cytohesin-interacting like (*cytip*)	DY700802	Forward	CGTCTTTGTGTGATGTGACG	0.997	104.5	151
		Reverse	GTCAAAGGGAGAATGCTTGC			
inhibitor of nuclear factor kappa-B kinase subunit alpha (*ikka*)	GE780687	Forward	AAAGACTGCACCCGACAAAC	0.992	93.8	111
		Reverse	TTGTGTCACCAAGGATGCTG			
mitogen-activated protein kinase 13 (*mapk13*)	NM_001141648	Forward	ACAGGCGATTGACCTTCTTG	0.999	95.7	133
		Reverse	ATATGGCTGTGGCTCAGGAC			
cd80	EG933501	Forward	CAGTGACACAGATCCCAGCA	0.996	97.3	107
		Reverse	GTTGTTAGTTTGCGCCTGTG			

dual specificity phosphatase 6 (*dusp6*)	NM_001165367	Forward<br>Reverse	AGAAAAGAGGGGAAGCGAAG<br>AGCCTTTCGTCGTGAAGCTA	0.994	90.7	167
dual specificity phosphatase 22-a (*dusp22a*)	NM_001140429	Forward<br>Reverse	CTGCGAGAGACATGGGAAAT<br>CAGACAGGATGTGGGTGATG	0.990	105.8	120
**Transcription factors**						
cAMP-responsive element modulator-like (*crem*)	CB508094	Forward<br>Reverse	GCTCTCTATGCAAGCCCTAGTC<br>AGACAAAATGCCCCCAGAG	0.992	98.4	110
interferon regulatory factor 7 (*irf7*)	BT045216	Forward<br>Reverse	CCAGTGCCACCAGTCCTAAT<br>GGTGATCTCCAAGTCCCAGA	0.999	94.5	105
cyclic AMP-dependent transcription factor ATF-3 (*atf3*)	BT059485	Forward<br>Reverse	CCAACTTCACTCCCCTGGTA<br>GGCTTCGGACTGTCTTTCTG	0.993	91.9	128
basic leucine zipper transcription factor, ATF-like 3 (*batf3*)	NM_001141610	Forward<br>Reverse	GAGCCTCTCTGTCCCATCAG<br>TGCATGGTATGGTGGCTATC	0.995	95.7	102
signal transducer and activator of transcription 1 (*stat1*)	DW551983	Forward<br>Reverse	GGTCCACACAAATCAACGTG<br>CTTTGCAGGGCCTTCTTCTT	0.998	97.1	154
**Immune effectors**						
ring finger protein 8, E3 ubiquitin protein ligase (*rnf8*)	NM_001173788	Forward<br>Reverse	CACGAGTCGGGAAAGATTTG<br>TATCATTAGGGGGCAGCTTG	0.996	100.3 [b]	116
CASP8 and FADD-like apoptosis regulator (*cflar*)	EG868690	Forward<br>Reverse	AGCTGGAGGAGAAGGACCAT<br>ATCTGGCTCCTGGATTCCTT	0.998	98.1	153
interferon-induced GTP-binding protein Mx (*mx-b*) [a]	NM_001139918	Forward<br>Reverse	ACGCACCACTCTGGAGAAAT<br>CTTCCATTTCCCGAACTCTG	0.995	93.2	184
optineurin (*optn*)	NM_001140289	Forward<br>Reverse	GAGCCAGCTAGAGCAGAGGA<br>CCCACCTCCTTAGTGACCAG	0.997	91.8	144
E3 ubiquitin-protein ligase herc3 (*herc3*)	DY693327	Forward<br>Reverse	GGTGACTGCTGGTCCTCATT<br>GGGTCACGAGTGCGTAGAAT	0.999	92.8	113
E3 ubiquitin-protein ligase herc6 (*herc6*)	EG915319	Forward<br>Reverse	CACCGGCTAACAGTGATGTG<br>GCACCCAGTTGTCTGAAGGT	0.998	92.6	128
interferon, gamma (ifng)	AJ841811	Forward<br>Reverse	CCGTACACCGATTGAGGACT<br>GCGGCATTACTCCATCCTAA	0.998	94.9	133
viperin	BT047610	Forward<br>Reverse	ACCATTTTACCCGACAGTGC<br>TCCCCAAGAAATCACCTCTG	0.995	103.2	183
beta-1 syntrophin (*sntb1*)	BT072489	Forward<br>Reverse	CACTCATGCCCAAAGCCTAT<br>ATAAGCCATGTTCGCCTCTG	0.997	95.3	194
cathepsin-L1-like (*ctsl1*)	DW575971	Forward<br>Reverse	TAGTTGTGGCTGCTGCTCTG<br>TAGGACTTGATGCCCTGGTC	0.997	86.8	199
cathepsin-f (*ctsf*)	NM_001140206	Forward<br>Reverse	TGCTGTGTTTTAGGCAGAGG<br>CAGGGGAACATAACGGACAC	0.995	98.7	122
**Normalizers**						
60S ribosomal protein 32 (rpl32)	BT043656	Forward<br>Reverse	AGGCGGTTTAAGGGTCAGAT<br>TCGAGCTCCTTGATGTTGTG	0.997	101.7	119
eukaryotic translation initiation factor 3 subunit D (*eif3d*)	GE777139	Forward<br>Reverse	CTCCTCCTCCTCGTCCTCTT<br>GACCCCAACAAGCAAGTGAT	0.995	101.4	105

[a] These transcripts were not present in the microarray significant gene list.

[b] The amplification efficiencies of these primers were determined using 4-point serial dilutions of cDNA.

The diet-responsive transcripts were selected from RP-identified gene lists. The pIC-responsive transcripts were selected from microarray-identified transcripts overlapping between SAM and RP in both dietary groups (783 DEP; see Fig. 2), except for *cd209d* (RP-identified in the FO5 group), and *stat1* and *irf7* (SAM-identified in both groups).
