# Supplementary material for: Transcriptome profiling of antiviral immune and dietary fatty acid dependent responses of Atlantic salmon macrophage-like cells
Source: BMC Genomics. 2017 Sep 8;18:706. doi: 10.1186/s12864-017-4099-2 (PMC5591513; doi:10.1186/s12864-017-4099-2)
Supplement: Supplementary file 5 — Hierarchical clustering analyses of samples based on of pIC-responsive transcripts overlapping between SAM- and RP-identified lists. (PDF 503 kb) [file 12864_2017_4099_MOESM5_ESM.pdf]

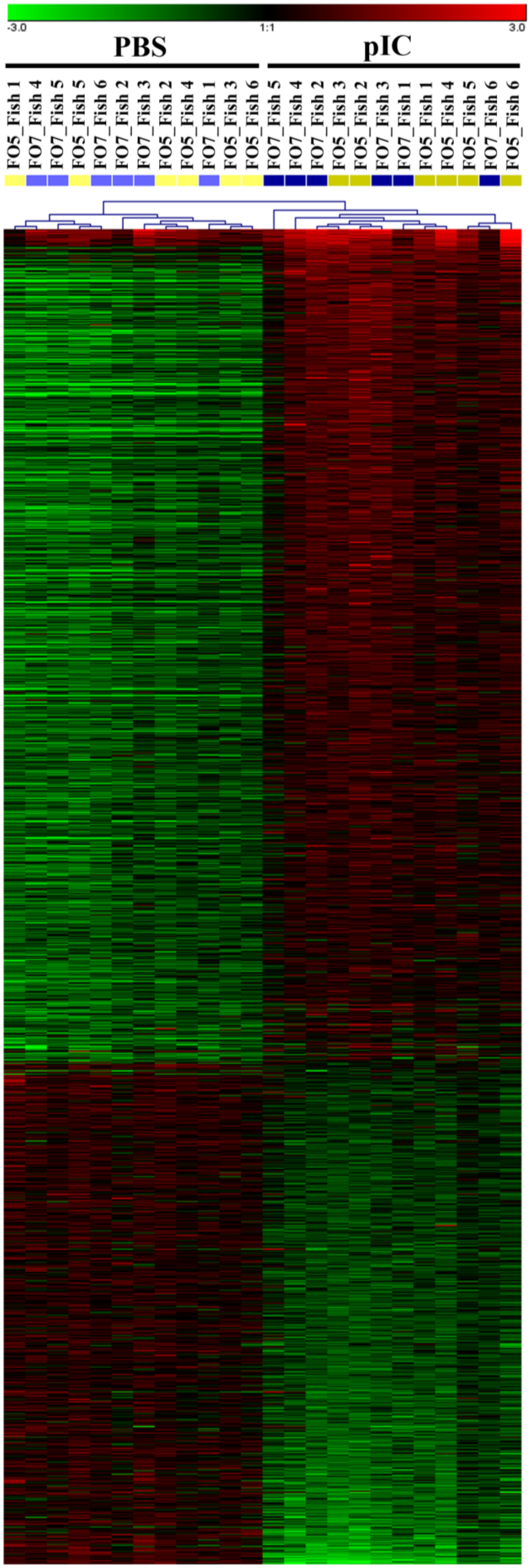

Supplemental Figure S2. Hierarchical clustering analyses of samples based on of pIC-responsive transcripts overlapping between SAM- and RP-identified lists. Coloured blocks at the top of the figure indicate the dietary and stimulation groups: light blue, PBS FO7; dark blue, pIC FO7; light yellow, PBS FO5; dark yellow, pIC FO5.
