# Supplementary material for: Transcriptome profiling of antiviral immune and dietary fatty acid dependent responses of Atlantic salmon macrophage-like cells
Source: BMC Genomics. 2017 Sep 8;18:706. doi: 10.1186/s12864-017-4099-2 (PMC5591513; doi:10.1186/s12864-017-4099-2)
Supplement: Supplementary file 6 — GO term annotation of pIC-stimulated transcripts in different dietary groups. (PDF 457 kb) [file 12864_2017_4099_MOESM6_ESM.pdf]

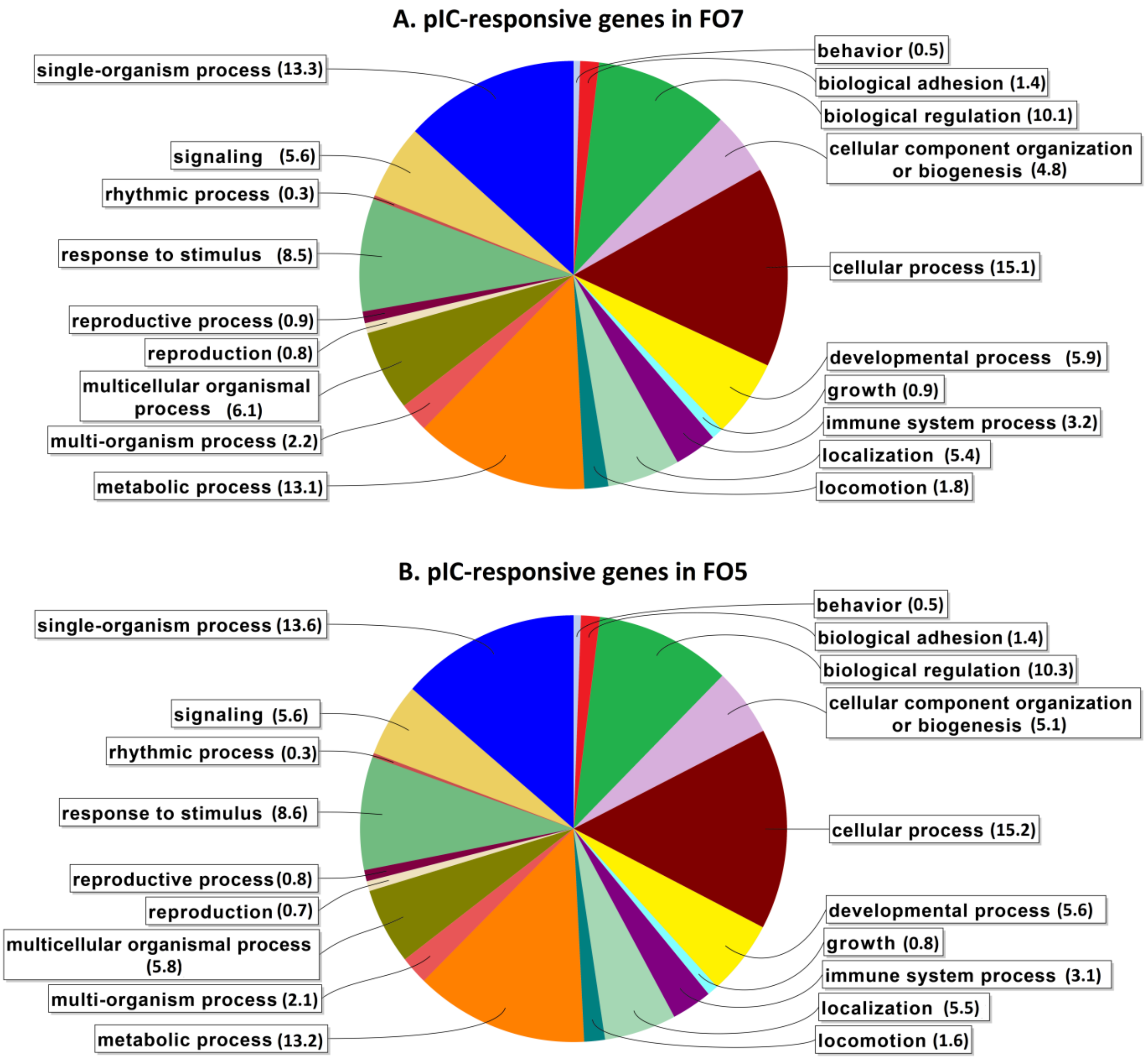

Supplemental Figure S3. GO term annotation of pIC-stimulated transcripts in different dietary groups. The charts illustrate the distribution of GO terms (Biological Process level 2) in pIC-responsive transcripts (i.e. overlapped between SAM- and RP-identified lists) of salmon MLCs in FO7 **(A)** and FO5 **(B)** groups. The numbers between brackets represent the percentage of probes with each GO annotation.
