# Supplementary material for: Transcriptome profiling of antiviral immune and dietary fatty acid dependent responses of Atlantic salmon macrophage-like cells
Source: BMC Genomics. 2017 Sep 8;18:706. doi: 10.1186/s12864-017-4099-2 (PMC5591513; doi:10.1186/s12864-017-4099-2)
Supplement: Supplementary file 7 — The enriched GO terms of pIC-responsive transcripts (overlap between SAM and RP analyses) within each dietary group (Fisher’s exact test, FDR < 0.05). (PDF 250 kb) [file 12864_2017_4099_MOESM7_ESM.pdf]

Supplemental Table S4. The enriched GO terms of pIC-responsive transcripts (overlap between SAM and RP analyses) within each dietary group (Fisher's exact test, FDR < 0.05)

GO ID	GO Term	Category [1]	Number of probes with GO [2] Test FO7	Test FO5	Over/Under
GO:0001739	sex chromatin	C	6	6	OVER
GO:0035102	PRC1 complex	C	6	6	OVER
GO:0044422	organelle part	C	150	222	UNDER
GO:0044446	intracellular organelle part	C	147	220	UNDER
GO:0043226	organelle	C	310	419	UNDER
GO:0032991	macromolecular complex	C	119	157	UNDER
GO:0043229	intracellular organelle	C	296	393	UNDER
GO:0044424	intracellular part	C	357	476	UNDER
GO:0031974	membrane-enclosed lumen	C	75	106	UNDER
GO:0070013	intracellular organelle lumen	C	73	105	UNDER
GO:0043233	organelle lumen	C	74	105	UNDER
GO:0044430	cytoskeletal part	C	18	30	UNDER
GO:0005856	cytoskeleton	C	30	50	UNDER
GO:0043234	protein complex	C	103	137	UNDER
GO:0044428	nuclear part	C	78	114	UNDER
GO:0043228	non-membrane-bounded organelle	C	85	117	UNDER
GO:0043232	intracellular non-membrane-bounded organelle	C	85	117	UNDER
GO:0005623	cell	C	423	566	UNDER
GO:0031981	nuclear lumen	C	68	96	UNDER
GO:0005622	intracellular	C	387	511	UNDER
GO:0044464	cell part	C	422	564	UNDER
GO:0043227	membrane-bounded organelle	C	302	409	UNDER
GO:0015630	microtubule cytoskeleton	C	13	25	UNDER
GO:0004842	ubiquitin-protein transferase activity	F	38	41	OVER
GO:0019784	NEDD8-specific protease activity	F	7	7	OVER
GO:0019787	ubiquitin-like protein transferase activity	F	38	41	OVER
GO:0004950	chemokine receptor activity	F	8	8	OVER
GO:0001637	G-protein coupled chemoattractant receptor activity	F	8	8	OVER
GO:0004896	cytokine receptor activity	F	11	14	OVER
GO:0004817	cysteine-tRNA ligase activity	F	5	5	OVER
GO:0071535	RING-like zinc finger domain binding	F	7	7	OVER
GO:0030911	TPR domain binding	F	5	5	OVER
GO:0001884	pyrimidine nucleoside binding	F	5	5	OVER
GO:0003917	DNA topoisomerase type I activity	F	4	4	OVER
GO:0004930	G-protein coupled receptor activity	F	23	31	OVER
GO:0004849	uridine kinase activity	F	3	5	OVER
GO:0016740	transferase activity	F	135	187	OVER
GO:0030429	kynureninase activity	F	2	3	OVER
GO:0051427	hormone receptor binding	F	14	18	OVER
GO:0019206	nucleoside kinase activity	F	5	7	OVER
GO:0008903	hydroxypyruvate isomerase activity	F	5	2	OVER
GO:0008270	zinc ion binding	F	85	89	OVER
GO:0046914	transition metal ion binding	F	95	103	OVER
GO:0016874	ligase activity	F	37	41	OVER
GO:0005525	GTP binding	F	39	40	OVER
GO:0032561	guanyl ribonucleotide binding	F	39	40	OVER
GO:0019001	guanyl nucleotide binding	F	39	40	OVER
GO:0003676	nucleic acid binding	F	112	152	UNDER
GO:0044822	poly(A) RNA binding	F	26	37	UNDER
GO:0003723	RNA binding	F	43	59	UNDER
GO:0008092	cytoskeletal protein binding	F	17	22	UNDER
GO:0097159	organic cyclic compound binding	F	215	272	UNDER
GO:1901363	heterocyclic compound binding	F	215	272	UNDER
GO:0070535	histone H2A K63-linked ubiquitination	P	9	9	OVER

GO:0036353	histone H2A-K119 monoubiquitination	P	6	6	OVER
GO:0033522	histone H2A ubiquitination	P	10	10	OVER
GO:0006423	cysteinyl-tRNA aminoacylation	P	5	5	OVER
GO:1901314	regulation of histone H2A K63-linked ubiquitination	P	6	6	OVER
GO:1901315	negative regulation of histone H2A K63-linked ubiquitination	P	6	6	OVER
GO:0033183	negative regulation of histone ubiquitination	P	6	6	OVER
GO:0071345	cellular response to cytokine stimulus	P	37	43	OVER
GO:1902915	negative regulation of protein polyubiquitination	P	6	6	OVER
GO:1900045	negative regulation of protein K63-linked ubiquitination	P	6	6	OVER
GO:1900044	regulation of protein K63-linked ubiquitination	P	6	6	OVER
GO:2000780	negative regulation of double-strand break repair	P	6	7	OVER
GO:0070682	proteasome regulatory particle assembly	P	5	5	OVER
GO:0070098	chemokine-mediated signaling pathway	P	9	9	OVER
GO:0034097	response to cytokine	P	40	50	OVER
GO:0042789	mRNA transcription from RNA polymerase II promoter	P	5	6	OVER
GO:1902914	regulation of protein polyubiquitination	P	6	6	OVER
GO:0045738	negative regulation of DNA repair	P	6	7	OVER
GO:0035518	histone H2A monoubiquitination	P	7	7	OVER
GO:0070534	protein K63-linked ubiquitination	P	11	11	OVER
GO:0045647	negative regulation of erythrocyte differentiation	P	5	5	OVER
GO:0009299	mRNA transcription	P	5	6	OVER
GO:0030219	megakaryocyte differentiation	P	9	9	OVER
GO:0035666	TRIF-dependent toll-like receptor signaling pathway	P	8	8	OVER
GO:0061299	retina vasculature morphogenesis in camera-type eye	P	5	6	OVER
GO:0071108	protein K48-linked deubiquitination	P	7	8	OVER
GO:0032434	regulation of proteasomal ubiquitin-dependent protein catabolic process	P	16	17	OVER
GO:0002756	MyD88-independent toll-like receptor signaling pathway	P	8	8	OVER
GO:0061298	retina vasculature development in camera-type eye	P	5	6	OVER
GO:0033182	regulation of histone ubiquitination	P	6	6	OVER
GO:0051131	chaperone-mediated protein complex assembly	P	5	5	OVER
GO:0060452	positive regulation of cardiac muscle contraction	P	4	4	OVER
GO:0042026	protein refolding	P	7	8	OVER
GO:0043174	nucleoside salvage	P	7	11	OVER
GO:0006954	inflammatory response	P	25	29	OVER
GO:0061304	retinal blood vessel morphogenesis	P	4	4	OVER
GO:0042221	response to chemical	P	140	180	OVER
GO:0071310	cellular response to organic substance	P	89	113	OVER
GO:0070887	cellular response to chemical stimulus	P	105	134	OVER
GO:0008655	pyrimidine-containing compound salvage	P	5	7	OVER
GO:0043097	pyrimidine nucleoside salvage	P	5	7	OVER
GO:2000779	regulation of double-strand break repair	P	6	7	OVER
GO:0044211	CTP salvage	P	3	5	OVER
GO:0071816	tail-anchored membrane protein insertion into ER membrane	P	1	5	OVER
GO:0043094	cellular metabolic compound salvage	P	9	12	OVER
GO:0045048	protein insertion into ER membrane	P	1	5	OVER
GO:0010138	pyrimidine ribonucleotide salvage	P	3	5	OVER
GO:0044206	UMP salvage	P	3	5	OVER
GO:0032262	pyrimidine nucleotide salvage	P	3	5	OVER
GO:0007186	G-protein coupled receptor signaling pathway	P	34	48	OVER
GO:0097053	L-kynurenine catabolic process	P	2	3	OVER
GO:0042182	ketone catabolic process	P	2	4	OVER
GO:0097309	cap1 mRNA methylation	P	2	3	OVER
GO:0010033	response to organic substance	P	108	140	OVER
GO:0032922	circadian regulation of gene expression	P	7	10	OVER
GO:0097052	L-kynurenine metabolic process	P	2	3	OVER
GO:0016574	histone ubiquitination	P	10	10	OVER
GO:0046686	response to cadmium ion	P	9	9	OVER
GO:0034138	toll-like receptor 3 signaling pathway	P	8	8	OVER

GO:0032436	positive regulation of proteasomal ubiquitin-dependent protein catabolic process	**P**	10	10	OVER
GO:0019915	lipid storage	**P**	7	6	OVER
GO:0002250	adaptive immune response	**P**	15	16	OVER
GO:0019221	cytokine-mediated signaling pathway	**P**	24	27	OVER
GO:0032480	negative regulation of type I interferon production	**P**	6	6	OVER
GO:0031057	negative regulation of histone modification	**P**	8	8	OVER
GO:0006282	regulation of DNA repair	**P**	9	10	OVER
GO:0007017	microtubule-based process	**P**	3	7	UNDER
GO:0071840	cellular component organization or biogenesis	**P**	145	205	UNDER
GO:0044085	cellular component biogenesis	**P**	53	76	UNDER
GO:0016043	cellular component organization	**P**	142	201	UNDER
GO:0060322	head development	**P**	10	15	UNDER
GO:0019725	cellular homeostasis	**P**	6	10	UNDER
GO:0022607	cellular component assembly	**P**	49	70	UNDER
GO:0007417	central nervous system development	**P**	15	20	UNDER
GO:0000226	microtubule cytoskeleton organization	**P**	3	6	UNDER
GO:0007399	nervous system development	**P**	47	66	UNDER
GO:0007420	brain development	**P**	10	14	UNDER
GO:0007018	microtubule-based movement	**P**	0	0	UNDER
GO:0003008	system process	**P**	24	25	UNDER
GO:0043009	chordate embryonic development	**P**	19	18	UNDER
GO:0006396	RNA processing	**P**	15	21	UNDER
GO:0009792	embryo development ending in birth or egg hatching	**P**	20	19	UNDER
GO:0008380	RNA splicing	**P**	4	6	UNDER
GO:0022613	ribonucleoprotein complex biogenesis	**P**	6	9	UNDER
GO:0044699	single-organism process	**P**	403	544	UNDER
GO:0016071	mRNA metabolic process	**P**	10	18	UNDER

[1] **C:** Cellular Component, **F:** Molecular Function and **P:** Biological Process

[2] Numbers of probes annotated with each GO term in pIC-responsive gene list overlapping between SAM and RP of each dietary group. Numbers highlighted in grey indicate a significant over- or under-representation (Fisher's exact test, FDR < 0.05) in the pIC-responsive gene list of the given dietary treatment, compared to the whole 44K salmon microarray. Total number of probes annotated with at least 1 GO term was 666 and 865 for FO7 and FO5 groups, respectively.
